# Genomic Impact of Whaling in North Atlantic Fin Whales

**DOI:** 10.1093/molbev/msac094

**Published:** 2022-05-05

**Authors:** Magnus Wolf, Menno de Jong, Sverrir Daníel Halldórsson, Úlfur Árnason, Axel Janke

**Affiliations:** Senckenberg Biodiversity and Climate Research Centre (BiK-F), Georg-Voigt-Strasse 14-16, Frankfurt am Main, Germany; Institute for Ecology, Evolution and Diversity, Goethe University, Max-von-Laue-Strasse. 9, Frankfurt am Main, Germany; Senckenberg Biodiversity and Climate Research Centre (BiK-F), Georg-Voigt-Strasse 14-16, Frankfurt am Main, Germany; Marine and Freshwater Research Institute, Fornubúðir 5, IS 220 Hafnarfjörður, Iceland; Department of Clinical Sciences Lund, Lund University, Sweden; Department of Neurosurgery, Skane University Hospital in Lund, Sweden; Senckenberg Biodiversity and Climate Research Centre (BiK-F), Georg-Voigt-Strasse 14-16, Frankfurt am Main, Germany; Institute for Ecology, Evolution and Diversity, Goethe University, Max-von-Laue-Strasse. 9, Frankfurt am Main, Germany; LOEWE-Centre for Translational Biodiversity Genomics (TBG), Senckenberg Nature Research Society, Georg-Voigt-Straße 14-16, Frankfurt am Main, Germany

**Keywords:** fin whales, bottleneck, genetic diversity, runs of homozygosity, whaling, mutational load, demography

## Abstract

It is generally recognized that large-scale whaling in the 19th and 20th century led to a substantial reduction of the size of many cetacean populations, particularly those of the baleen whales (*Mysticeti*). The impact of these operations on genomic diversity of one of the most hunted whales, the fin whale (*Balaenoptera physalus*), has remained largely unaddressed because of the paucity of adequate samples and the limitation of applicable techniques. Here, we have examined the effect of whaling on the North Atlantic fin whale based on genomes of 51 individuals from Icelandic waters, representing three temporally separated intervals, 1989, 2009 and 2018 and provide a reference genome for the species. Demographic models suggest a noticeable drop of the effective population size of the North Atlantic fin whale around a century ago. The present results suggest that the genome-wide heterozygosity is not markedly reduced and has remained comparable with other baleen whale species. Similarly, there are no signs of apparent inbreeding, as measured by the proportion of long runs of homozygosity, or of a distinctively increased mutational load, as measured by the amount of putative deleterious mutations. Compared with other baleen whales, the North Atlantic fin whale appears to be less affected by anthropogenic influences than other whales such as the North Atlantic right whale, consistent with the presence of long runs of homozygosity and higher levels of mutational load in an otherwise more heterozygous genome. Thus, genome-wide assessments of other species and populations are essential for future, more specific, conservation efforts.

## Introduction

### Fin Whales and Whaling

Fin whales (*Balaenoptera physalus*) are a species of cosmopolitan rorquals (*Balaenopteridae*) within the group of baleen whales (*Mysticeti*) (Edwards et al. 2015; Aguilar and García-Vernet 2017). They are among the largest species on Earth and are known for migrating seasonally between low latitude breeding and high latitude feeding grounds (Silva et al. 2013; Lydersen et al. 2020). Despite their global occurrence, fin whales rarely cross the equatorial regions, and their distribution is therefore defined by the equator and major landmasses (Edwards et al. 2015). As these restrictions have existed for long periods of time, they have made it possible to differentiate fin whales into distinct populations and subspecies based on both phenotypic and genotypic features (Lockyer and Waters 1986; Edwards et al. 2015; Archer et al. 2019).

Fin whales have been subjected to large-scaled whaling since first industrialized operations in the 1870s (Aguilar and García-Vernet 2017). In 1904, a first local over-exploitation was reached in the waters of northern Finnmark (Norway) after which the local whaling industry was forced to either switch targets to, for example, the minke whale, *Balaenoptera acutorostrata* or move to other locations (Tønnessen and Johnsen 1982). From there on, whaling expanded around the globe with reoccurring over-exploitations and relocations of industry infrastructure until catch rates peaked between 1925 and 1960 with records of ∼30,000 individuals taken annually (Aguilar and García-Vernet 2017). In the 1960s, 1970s and 1980s, increasingly strict whaling limitations were introduced and later a complete moratorium was enforced by the International Whaling Commission due to dwindling stock sizes and imminent extinctions of several baleen whale species (Smith 1984). This led to a noticeable recovery of fin whale population sizes and current estimates add up to ∼90,000–100,000 individuals worldwide of which 40,000–60,000 are allocated to the North Atlantic (Aguilar and García-Vernet 2017; Hansen et al. 2019). A recent survey conducted in the North Atlantic area around Icelandic waters sized up 30,000 individuals for this area alone (Pike et al. 2019). Eventually, the recent recovery led to a change in the threat status of the IUCN red list from “endangered” to “vulnerable” (Cooke 2018).

### Genetic Diversity and Conservation

Population survivability is not dependent on census sizes only, but is also shaped by genetic diversity, which is a proxy of the adaptive potential and hence long-term survival of a species (Booy et al. 2000), a circumstance which does not necessarily coincide with abundance (Coates et al. 2018). There are numerous examples of species with relatively high present-day census sizes but low genetic diversity such as the Madagascar fish-eagle, the brown hyena or the narwal (Johnson et al. 2009; Westbury et al. 2018, 2019). Despite these examples, it is commonly assumed that populations with low genetic diversity are more vulnerable to extinction than others because in cases of rapidly changing environments, a low genetic diversity might result in a decreased adaptive potential and hence a lowered reproduction rate and increased mortality (Reed and Frankham 2003; Spielman et al. 2004a; Frankham 2005).

In cases of low genetic diversity and effective population size (*N*_e_), inbreeding may cause an accumulation of homozygous recessive mutations that eventually affect the fitness of a species due to their deleterious effects (Tanaka 2000). The abundance of deleterious mutations, the so-called mutational load, might further increase, because of a reduced efficiency of purifying selection (Ohta 1973). This reciprocal relationship between genetic diversity, inbreeding, mutational load and fitness is widely known as the “extinction vortex” and has been studied in detail, both on theoretical grounds and to guide conservation practices (Charlesworth and Willis 2009; Kimura et al. 1963; Leimu et al. 2006; Spielman et al. 2004b; Tanaka 2000).

Genome-wide studies addressing these topics are still rare, despite their promise to yield comprehensive conclusions on the genetic diversity and the general fitness of a population (Bortoluzzi et al. 2020; von Seth et al. 2021; van der Valk et al. 2019b; Westbury et al. 2018). Moreover, inclusive studies addressing inbreeding or mutational load require well assembled and annotated genomic data or genome information from numerous individuals. Until recently, computational and economic limitations have hindered a common application of whole-genome-sequencing data in conservation biology. However, since sequencing costs are steadily decreasing and computational power is increasing, these limitations are disappearing, enabling a broad and large-scale application of conservation-genomic analyses.

### Objectives

In this study, we assess the genomic consequences of industrial whaling for a North Atlantic fin whale population over a time period of three decades, spanning approximately one full generation time of the species. We sequence the genomes of 51 fin whale individuals which were sampled around Iceland in 1989, 2009, and 2018 and provide a new high-quality reference genome assembly for the species. Genome data is used to model the demographic past of the population and to quantify genome-wide heterozygosity as a measure for genetic diversity. To analyze potential genetic consequences, inbreeding factors are calculated based on the distribution of runs of homozygosity (ROH) and the mutational load is estimated by identifying the abundance of potential deleterious mutations. These results are compared to a broad selection of other baleen whale species that experienced different magnitudes of whaling (Tønnessen and Johnsen 1982). With this study we aim to present an overview of the genetic variability in North Atlantic fin whales and demonstrate the need for comprehensive, genome-wide data to assess the genetic impact and consequences of a bottleneck caused by extensive hunting.

## Results

### Genome Characteristics and Completeness

A high-quality reference genome for the fin whale (*Balaenoptera physalus*) was assembled to a total length of 2.412 Gbp with a contig N50 of 24.9 Mbp and a L50 of 27 contigs (supplementary table S2, Supplementary Material online). The longest contig has a length of 91.5 Mbp and the GC content of the total assembly is 40.8%. Completeness analyses of three different busco datasets, namely of the clades *Cetartiodactyla*, *Laurasiatheria,* and *Mammalia*, returned estimates of 83.4%, 90.5%, and 91.2% complete core gene sets, respectively. Repeatmasking identified a total repeat coverage of 41.8% mainly composed of retroelements (38.1%) (supplementary table S3, Supplementary Material online). The annotation of the fin whale using the transcriptome data of the minke whale (Yim et al. 2014) resulted in 17,307 complete transcripts. A functional annotation using interprocan v5 (Jones et al. 2014) allocated potential functions for 17,152 genes, corresponding to more than 99% of all found transcripts.

### Demography

**Fig. 1.**
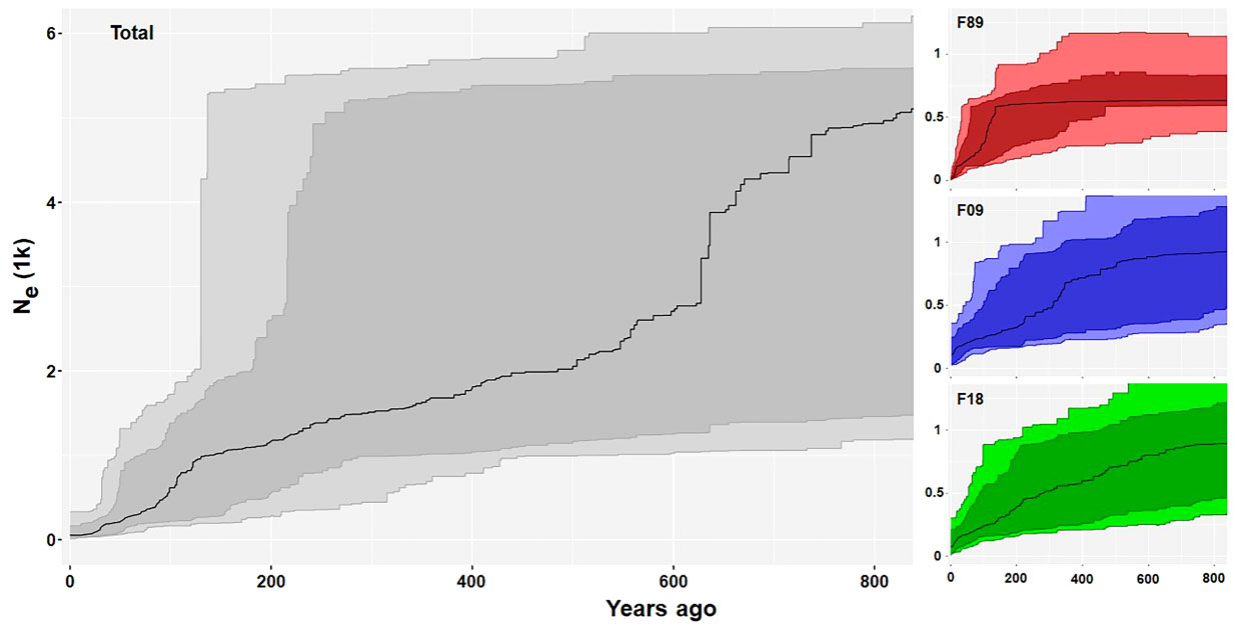
Changes in *N*_e_ over the last 800 years for all analyzed fin whales combined (total, gray), or for the three fin whale cohorts 1989 (F89, red), 2009 (F09, blue) and 2018 (F18, green) separately, estimated by stairway  plot v2 (Liu and Fu 2020). Plots were scaled using a mutation rate of 1.54 × 10^−9^ per site per generation and a generation time of 25.9 years. All models show a wide variety of signals, including a steep reduction in *N*_e_ around 100–150 years ago (upper 2.5% confidence interval) or only a minor gradual decline over the past 800 years (lower 2.5% confidence interval). A bottleneck pattern is more prominent in the 1989 cohort, whereas a declining pattern is more prominent in the more recent cohorts.

We modeled the demographic history of the North Atlantic fin whale population using stairway plot v2 (Liu and Fu 2020) which, based on the folded site frequency spectrum (fSFS, supplementary fig. S2, Supplementary Material online), estimates changes in the effective population size (*N*_e_) over time (fig. 1). Changes in *N*_e_ over the past 800 years follow a similar trajectory for the combined number of individuals as well as for the three cohorts separately suggesting a slow and steady decline for most of the modeled time period. All models depict a wide variety of patterns with some showing a steep short-lived drop of 80% ∼100–150 years ago while others show only minor changes. The drop in *N*_e_ is more prominent in the estimation of the combined data and the 1989 cohort compared with the estimations of the two more recent cohorts of 2009 and 2018. Nevertheless, signals of population reduction are obvious in the confidence intervals of all models. To verify these results, we simulated fSFS given a wide range of demographic scenarios using SLiM (supplementary fig. S3, Supplementary Material online, Haller and Messer 2019). Log-likelihoods of observing the empirical fSFS given one of the simulated fSFS (supplementary fig. S4, Supplementary Material online) were then calculated for each scenario and revealed that a severe population reduction leads to a more similar fSFS compared with scenarios without such an event (supplementary table S4, Supplementary Material online). Doing so also revealed that migration from a non-affected population to a population with bottleneck always weakened the performance of the respective simulation.

In addition, a pairwise sequentially Markovian coalescent (PSMC) analysis (Li and Durbin 2011) was used to model changes of *N*_e_ between 1 million (Mya) and 10,000 (kya) years ago (supplementary fig. S5, Supplementary Material online). Similar to Árnason et al. (2018), the population size first decreased over a period of 1–300 kya, then increased between 300 kya and 200 kya, before decreasing again slightly. However, the variety of patterns recorded among different individuals suggest that the demographic past could have been more complex because so6me individuals indicate more stable population trajectories compared with others.

### Heterozygosity and Genetic Diversity

**Fig. 2.**
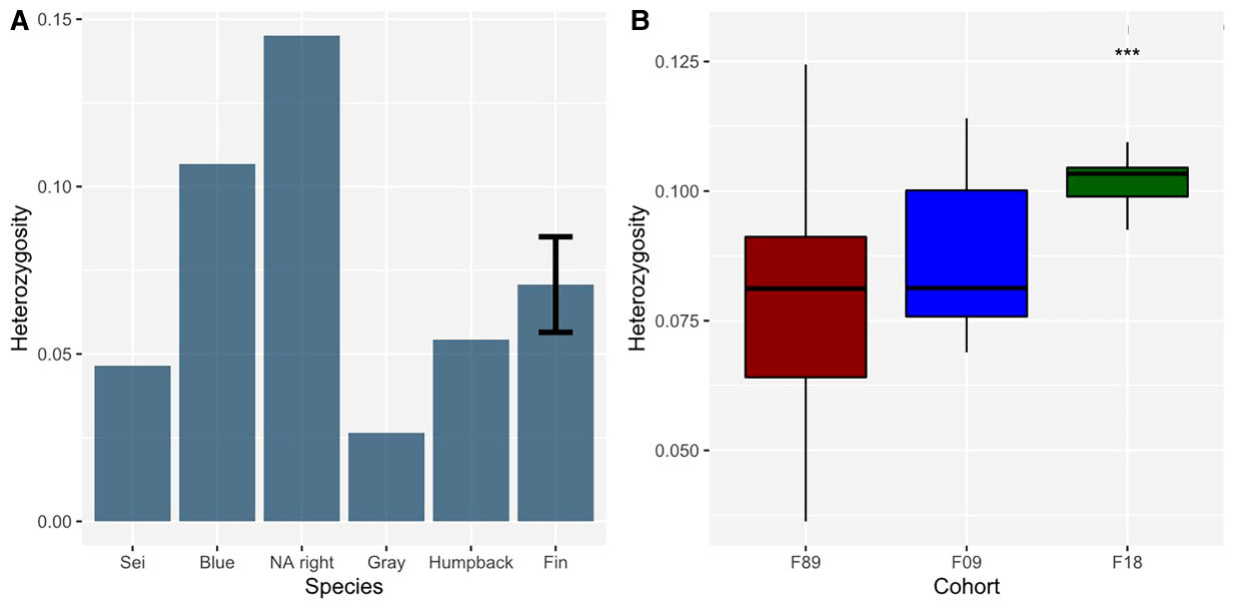
Genome-wide heterozygosity, (*y*-axis) in percent of heterozygous sides in the SNP and SNV data sets, respectively. (*A***)** Heterozygosity within the baleen whale SNV data set using the bowhead whales as a reference. Fin whales show a moderate heterozygosity of around 0.07%. The blue whale and North Atlantic right whale individuals had a higher He (0.11% and 0.15%), whereas sei, gray, and humpback whales were less heterozygous (0.05%, 0.03%, and 0.05%). (*B***)** Box plot of the He distribution between three fin whale cohorts sampled in 1989 (red), 2009 (blue), and 2018 (green), respectively. A slight increase in He over the three cohorts was observed, with individuals sampled in 2018 differing significantly (*P* = 0.0051) from the 1989 and 2009 cohorts.

Genetic diversity for the study population and for other baleen whale species was estimated by genome-wide heterozygosity (He), nucleotide diversity (*π*), Tajima’s *D,* and Watterson’s Θ (table 1). Mean levels of heterozygosity within the fin whale population differed significantly (ANOVA f(2) = 6.1, *P* = 0.005) between the three cohorts, equaling, respectively, 0.08%, 0.09%, and 0.1% (fig. 2*B*). The variance within the population decreased over the three sampling periods from 2.1e−4 in 1989 to 4.6e−5 in 2018. The observed nucleotide diversity *π* equaled respectively 0.216, 0.23, and 0.23, whereas Tajima’s *D* equaled 0.036, 0.036, and 0.029.

**Table 1.** Statistics for Genetic Diversity, Inbreeding, and Mutational Load Inferred for Two Different Whole-Genome Data Sets, One Including 51 Fin Whale Genomes and One Including Different Available Baleen Whale Genomes.

Statistic	Cohort			Species					
	F89	F09	F18	Fin	Sei	Blue	Gray	Humpback	NA right
Mean He	0.079	0.088	0.102	0.068	0.045	0.118	0.026	0.05	0.141
Mean (*π*)	0.252	0.255	0.261	0.099	—	—	—	—	—
Tajima’s *D*	0.036	0.023	0.029	−0.008	—	—	—	—	—
Watterson Θ	0.216	0.232	0.232	0.107	—	—	—	—	—
*F*_H_	−0.007	−0.038	−0.07	—	—	—	—	—	—
*F*_ROH_ (>1 Mbp)	0.019	0.004	0.006	0.081	0.046	0.195	0.027	0.050	0.427
# LoF Mutations	258	256	289	1108	1443	1685	1792	228	1094
Mutational Load	1.6e^−04^	1.1e^−04^	1.4e^−04^	2e^−04^	1.8e^−04^	1.9e^−04^	2.3e^−04^	2.1e^−04^	2e^−04^
# He LoF	157	157	187	119	141	235	77	32	344
# Ho LoF	101	100	102	989	1302	1450	1715	196	750

He, heterozygosity.

Ho, homozygosity.

*π*, nucleotide diversity.

*F*_H_, inbreeding coefficient following Kardos et al. (2015).

*F*_ROH_(>1 Mbp), inbreeding factor based on runs of homozygosity over 1 Mbp in percent.

# LoF, mean number of loss of function mutations.

NA right, North Atlantic right whale.

The 51 fin whale individuals are further differentiated into three cohorts based on their sampling year (F89 = 1989, F09 = 2009, and F18 = 2018). Some statistics are not applicable (—) for all species because most other baleen whales were represented by a single individual per species.

Compared with other whales, our combined fin whale data set mapped to the bowhead whale identified an average genome-wide heterozygosity of 0.07% while other baleen whales were found to have higher or lower proportions (fig. 2*A*). We found lower genome-wide heterozygosity in the humpback whale (0.05%), the sei whale (0.05%), and the gray whale (0.03%). In contrast, the blue whale (0.12%) and the North Atlantic right whale (0.14%) exhibited higher levels of heterozygosity.

### Inbreeding and ROH

Genome-wide signs of inbreeding were studied by two different approaches. First, we measured inbreeding in the three cohorts by comparing the numbers of expected heterozygous sites against the observed number (inbreeding factor *F*_H_) using sambaR’s “calckinship” function, following the definition of Kardos et al. (2015) (table 1, supplementary fig. S6, Supplementary Material online). Inbreeding factors *F*_H_ were slightly negative in all three fin whale cohorts. Although a *F*_H_ of −0.007 in 1989 identifies a nearly expected number of homozygous genotypes, a decrease to −0.038 in 2009 and to −0.070 in 2018 suggests a slight excess in heterozygous genotypes compared with expected values.

**Fig. 3.**
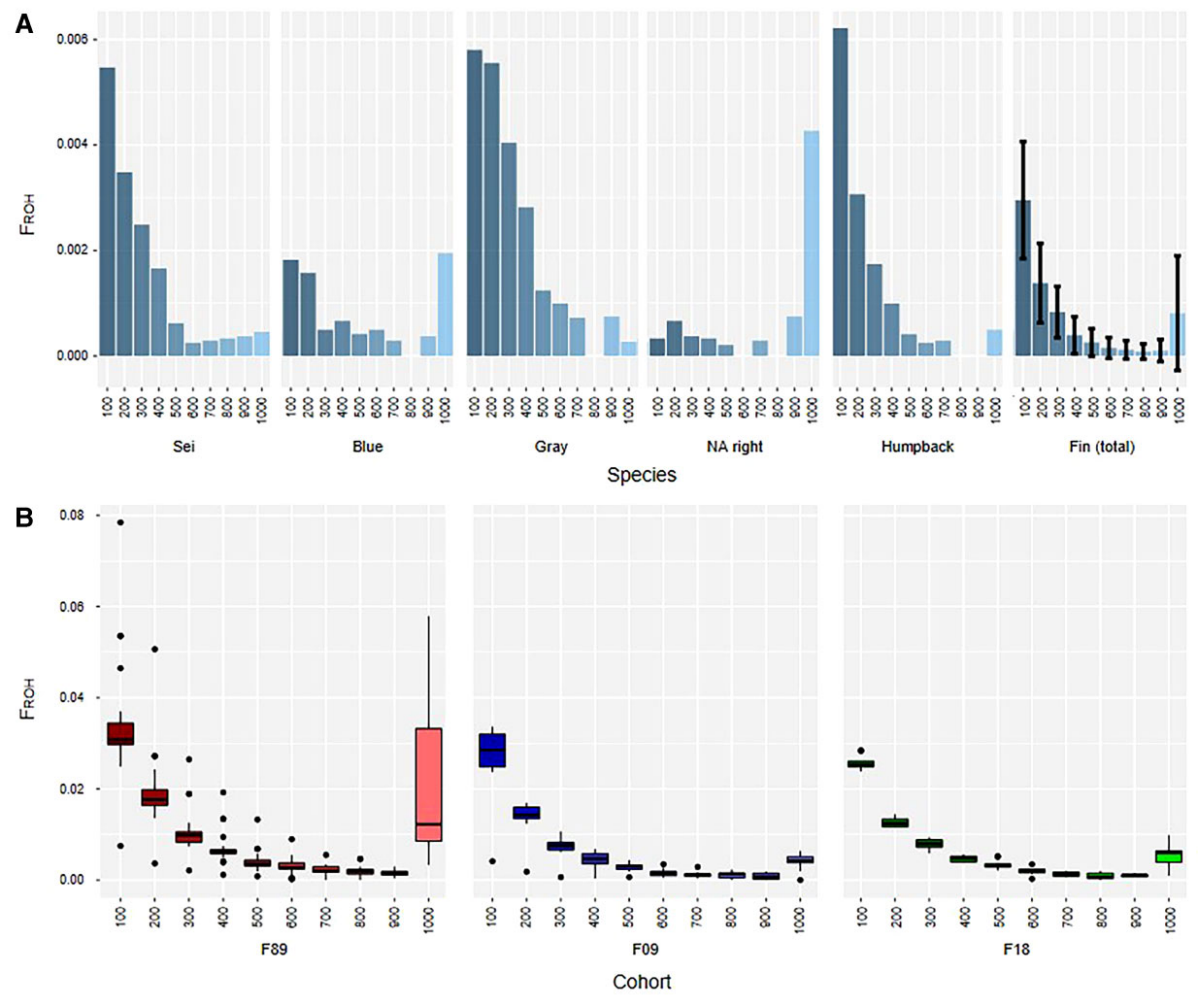
Inbreeding factors (*F*_ROH_) based on the genome coverage of run of homozygosity (ROH) between different minimal lengths cutoffs of ROH: 100 kbp to 1 Mbp (*x*-axis in 100 kbp steps). (*A*) Comparison of *F*_ROH_ among genomes of different baleen whale species and the 51 fin whales. While sei, gray and humpback whale have similar patterns of gradually decreasing inbreeding coefficients in their ROH length bins from 0.6% to <0.1%, varying patterns of high *F*_ROH_ (∼0.2–0.4%) in the >1 Mbp bin and low *F*_ROH_ (<0.1–0.2%) in the 100 kbp bin were found in the genomes of blue and North Atlantic right whale indicating more recent inbreeding events. The fin whale population shows an overall similar pattern to the sei, gray and humpback whale but with potentially higher inbreeding coefficients on the >1 Mbp length bin in some of the individuals (∼0.2%). (*B*) Comparison of *F*_ROH_ between the three different fin whale cohorts 1989 (red), 2009 (blue), and 2018 (green). We found four outlier individuals with higher or lower inbreeding coefficients and a significantly higher amount of long >1 Mbp ROH (*F*_ROH_ up to 0.6%) in the 1989 cohort. In the other two cohorts, only the same gradually decreasing pattern of inbreeding coefficients was identified.

We calculated inbreeding factors (*F*_ROH_) based on ROH as the coverage of runs exceeding a defined size cutoff, beginning with 100 kbp and increasing stepwise to 1 Mbp (fig. 3*B*). A similar gradually decreasing pattern of inbreeding factors in the defined length bins was identified in all three cohorts, beginning with an average of ∼3% in the 100–200 kbp bin and declining to an average of ∼1% in the >1 Mbp bin. Apart from this general pattern, four outlier individuals were noticed in the 1989 cohort, featuring more or less ROH in most of the bin. Furthermore, significantly more ROH of the longest category (>1 Mbp) were found within the 1989 cohort compared to both other cohorts (table 1, supplementary fig. S8, Supplementary Material online), indicating that some individuals within this cohort experienced more recent inbreeding.

When compared with other baleen whales (fig. 3*A*), fin whales featured the same gradual decrease as identified for the individual cohorts (total *F*_ROH_: 0.7%). Similar distributions but with generally higher inbreeding coefficients were noticed for the sei (total *F*_ROH_: 1.5%), gray (total *F*_ROH_: 2.2%), and humpback whale (total *F*_ROH_: 1.3%). In the blue and North Atlantic right whales, however, divergent distributions without this gradual decrease were found. In those individuals, low or lowered numbers of short ROH were recorded, whereas long ROH (>1 Mbp) were much more frequent (table 1). Especially in the North Atlantic right whale, long ROH accounted for more than half (0.4%) of the total inbreeding coefficient of 0.7%. Long ROH in the blue whale genome made up 0.2% of the total 0.8% *F*_ROH_. Furthermore, we found a negative correlation trend between the total *F*_ROH_ coefficients and genome-wide heterozygosity (fig. 5*A*) within the combined data set. Inbreeding factors *F*_H_ and genome-wide heterozygosity showed a slight positive correlation trend.

### Mutational Load

**Fig. 4.**
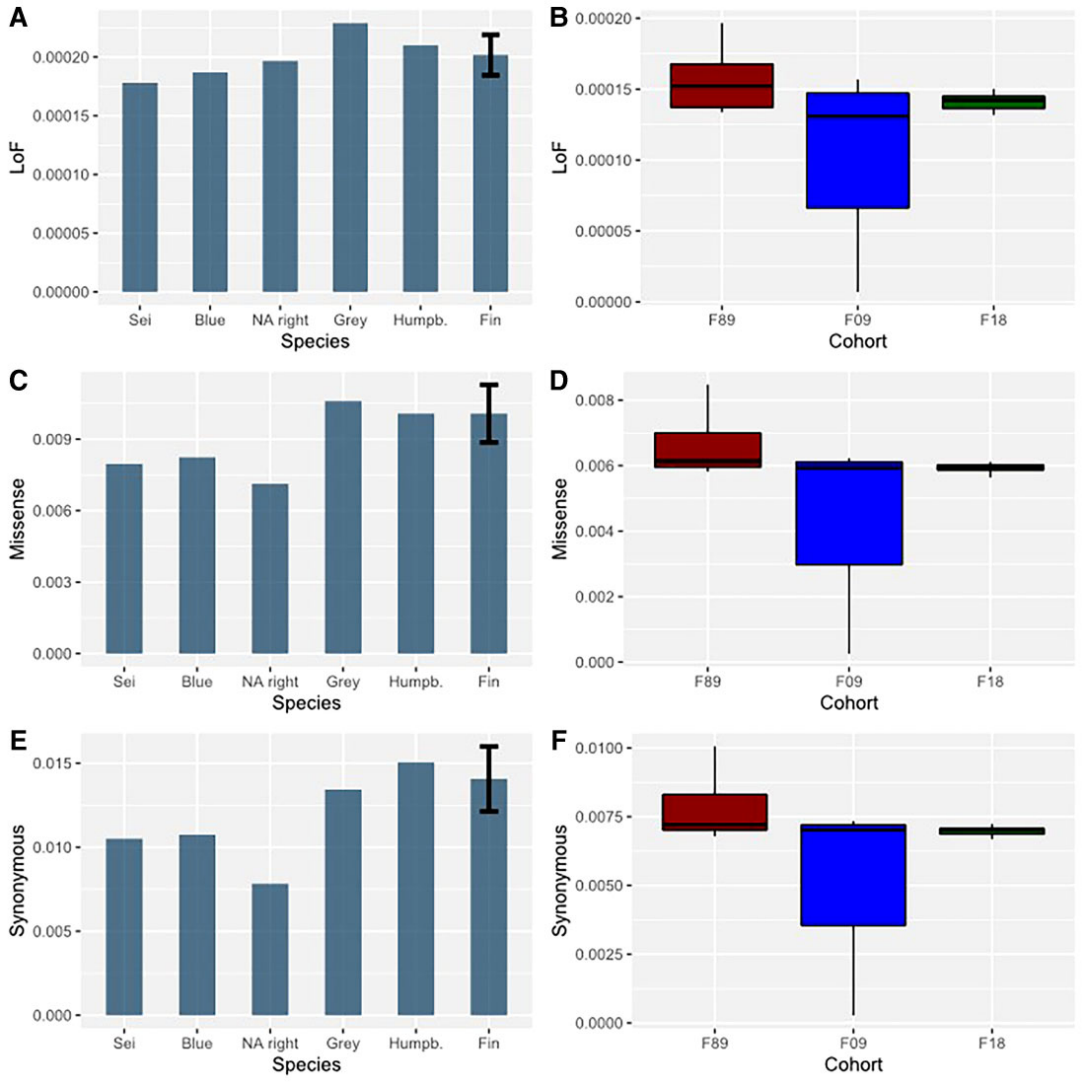
Abundances of three categories of functional mutations (LoF, missense and synonymous), measured as their relative proportion compared with the total number of SNPs or SNVs, respectively. Within the baleen whale data set (*A*, *C* and *E*), fin whales show a relatively high abundance of mutations in every category. By contrast, the North Atlantic right whale has a comparable high number of LoF mutations relative to the respective abundances of missense and synonymous mutations. No significant differences were found in the 51 fin whale genomes between the three sampling years 1989 (red), 2009 (blue), and 2018 (green) (*B*, *D*, and *F*). Fin whales from 1989 always have the most mutations in every category while whales from 2009 always have the least number of mutations. The overall variation in the 2018 cohort was, in general, lower compared with the other two cohorts. Yet, all cohorts had a similar medium number of mutations in every category.

Mutations with a potentially negative fitness impact were identified by annotating our single nucleotide polymorphism (SNP) and single nucleotide variants (SNV) datasets based on the genome annotation of this study or from the bowhead whale genome resource (Keane et al. 2015). Using SNPeff v4.3 (Cingolani et al. 2012), an average of 1.26% of our fin whale datasets and 2.4% of our baleen whale dataset were labeled as functional mutations and sorted into the three categories: *synonymous, missense and loss of function* (LoF). Based on these annotations, we found on average 0.69% synonymous mutations, 0.58% missense mutations, and 0.01% LoF mutations in the fin whales SNP data while we identified 1.37% synonymous, 0.99% missense, and 0.02% LoF mutations in the SNV data of all baleen whales (fig. 4*A*). Between the different cohorts, no significant differences were observed in the three functional categories (fig. 4*B*). Fin whales from the 1989 cohort featured the most annotated mutations in every category, but individuals from the 2018 cohort possessed a slightly higher mean number of LoF mutations. All three cohorts had a similar number of variants in a homozygous state (∼100), yet the 2018 cohort showed on average 30 heterozygous LoF mutations more compared with the other two.

Among other baleen whale species, only minor differences in the mutational load were identified. However, the North Atlantic right whale seems to have a slightly increased proportion of LoF mutations respective to the proportions of other categories, but none of these differences were significant (fig. 4*A*, *C*, and *E*).

**Fig. 5.**
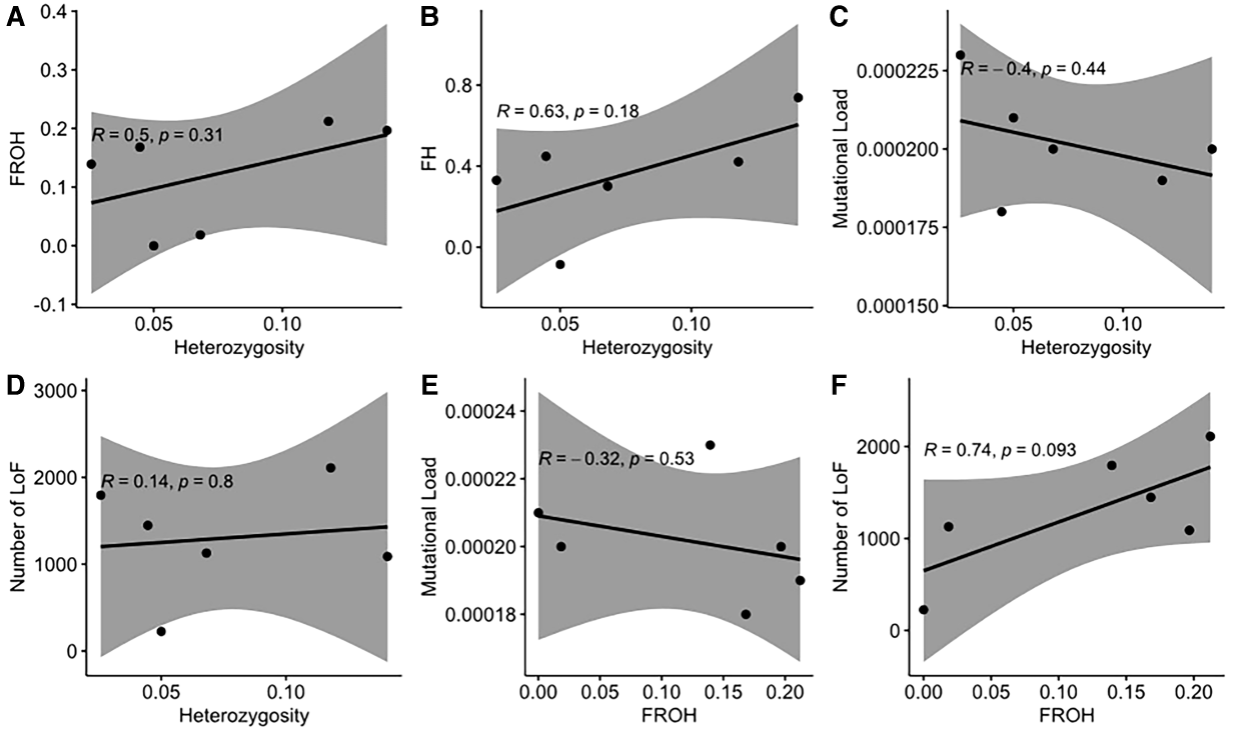
Cross correlation analysis between genome-wide heterozygosity, inbreeding and mutational load showing the potential correlations between heterozygosity and *F*_ROH_ as well as *F*_H_ (*A* and *B*) and between the relative frequency of LoF mutations (*C* and *E*) or total number of LoF mutations (*D* and *F*) against heterozygosity or *F*_ROH_. We identified a lack of correlations between nearly all parameters. Only heterozygosity and *F*_ROH_ have a non-significant negative trend towards each other as indicated by a *R* = −0.81 and *P* = 0.053. Neither the relative frequency nor the total number of LoF mutations showed any such trends.

Finally, no significant correlations were observed between the mutational load or the total number of LoF mutations to either genome-wide heterozygosity or the inbreeding coefficient based on ROH (fig. 5*C*–*F*). The relationship between mutational load and heterozygosity shows a more negative trend, and the relationship between mutational load and inbreeding shows a more positive trend.

### Population Structures

**Fig. 6.**
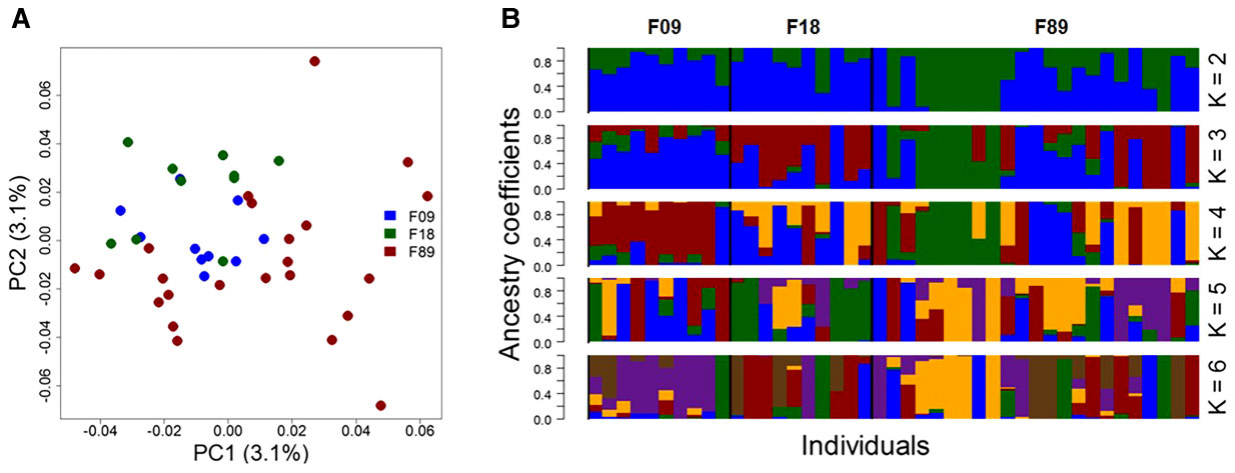
Population structure analyses of fin whales sampled in Icelandic waters in 1989 (red), 2009 (blue), and 2018 (green), respectively. (*A***)** PCoA identified only one major fin whale population. (*B*) The admixture-like analysis (colors indicate clusters inferred by the algorithm) resulted in no clear structure, indicating free exchange of genetic material in this population over all three cohorts.

The two conducted population structure analyses identified no sub-structures within the sampled fin whale individuals (fig. 6). The admixture analysis conducted with the lea package (Frichot et al. 2015) produced random signals of admixture that affected all individuals regardless of the assumed K. The principal coordinate analysis (PCoA) found only one cluster (PC1 depicts 3.1% variance, PC2 3.1%).

## Discussion

In this study, we analyzed the genome-wide diversity, inbreeding, mutational load and the demographic history of a North Atlantic fin whale population and other baleen whales that might have been affected by large-scale whaling in the past. Modeling the recent demographic history of the fin whale population using the site frequency spectrum, we were able to quantify the reduction of the population a century ago. Thus, the exploitation of fin whales left a noticeable signature in their genomes that coincides with maximum hunting pressure on the species (Tønnessen and Johnsen 1982). It is uncertain if this signature is characteristic for the North Atlantic population, or, more likely, is found in all fin whales due to the worldwide exploitation of this species in the past (Tønnessen and Johnsen 1982; Aguilar and García-Vernet 2017).

Despite the genomic and documented impact of whaling on the effective population size (Tønnessen and Johnsen 1982), North Atlantic fin whales do not show signs of genomic consequences that would affect their overall genetic fitness. The population features a moderate level of heterozygosity and neither excessively long ROH nor a pronounced excess of loss of function mutations when compared with other baleen whales. Instead, found genetic consequences are relatively weaker compared with other baleen whales that were proportionally more affected by whaling like the blue whale (Tønnessen and Johnsen 1982) and are comparable with baleen whales that were hunted on a similar or lower scope like the sei whale and the humpback whale (Tønnessen and Johnsen 1982), respectively. Other genomic studies addressing more threatened or potentially extinct species and populations such as the Grauer’s gorilla, the Scottish killer whale, or the Malay Peninsula rhinoceros (van der Valk et al. 2019a; Foote et al. 2021; von Seth et al. 2021) show more pronounced genomic consequences, such as substantial genome coverage of ROH longer than 1 Mbp and a significant increase of loss of function mutations. Therefore, our study does not support a molecular threat of the North Atlantic fin whale population. Instead, we suspect the levels of genetic diversity, inbreeding, and mutational load to be the result of a long-lasting demographic pattern with minor, natural fluctuations with limited impact on the genomes of the population.

The fact that the population bottleneck inflicted by whaling was short-lived and relatively recently might explain the lack of negative genetic consequences in fin whales. In theory, negative effects could still appear in later generations, because, for example, the mutational load is expected to not increase drastically unless the bottleneck is persistent (Teixeira and Huber 2021). Given the expected generation time of about 26 years for fin whales (Taylor et al. 2007), ∼4–6 generations have passed since whaling peaked in 1915. However, population-genetic theory and empirical findings suggest that a bottleneck of a few generations can cause long ROH and decreased levels of heterozygosity (Nei et al. 1975; Gurgul et al. 2016). Both effects were not observed in the presented sampling. Instead, we observed a significant increase in heterozygosity over the studied 30-year period and only few regions of long ROH.

An alternative explanation might be a genetic exchange with other populations or even species, which may be supported by signals of no reduction in the demographic analysis (fig. 1) and by the occurrence of outlier individuals in the ROH analysis of the 1989 cohort (fig. 3). Such a genetic exchange is often desired in conservation management plans by adding individuals from a stable population to a threatened one and is often referred to as a “genetic rescue” (Frankham 2015). Seven stocks of fin whales are currently assumed by the North Atlantic Marine Mammal Commission for the North Atlantic (NAMMCO 2005) and at least two genetically and morphologically distinct populations have been verified (Bérubé et al. 1998). It is possible that genetic exchange between one of those populations and the Icelandic fin whales weakened the genetic consequences of extensive hunting. In addition, genetic exchange between different ocean basins is evident from the analyses of mitogenome and SNP data (Archer et al. 2019). Although genetic exchange cannot be excluded, the relative genetic uniformity between most fin whale individuals suggests that genetic exchange between different populations is rare and may not explain the overall lack of negative genetic consequences in the fin whale population. Furthermore, our demographic simulations resulted in lower model performance when including migration from a non-affected population to a bottleneck population, which either shows that all fin whale populations were impacted by whaling or that migration happens on a small scope.

Introgression from blue whale genomes might be another possibility and may also have contributed to the genetic diversity of North Atlantic fin whales. However, blue whales are potentially more affected by census size depletion and show more signs of genetic consequences compared to the here analyzed fin whales (figs. 2–4). Furthermore, it seems that introgression between both species is unidirectional from fin to blue whale (Jossey et al. 2021; Pampoulie et al. 2021).

Despite the substantial impact of whaling on the effective population size of North Atlantic fin whales, the apparent lack of other genomic consequences challenges the common concern of a fatal over-exploration of fin whales by 19th and 20th century whaling. Instead, it could be possible that the bottleneck inflicted by Icelandic whaling never reached a duration or scale that would have triggered widespread genomic changes. Iceland was involved in industrial whaling for three decades between 1883 and 1915 (Tønnessen and Johnsen 1982). During this time, about 60,000 captured baleen whales have been reported in the complete Northern Atlantic. It can be expected that a major proportion of these catches were fin whales, however, estimating total numbers is problematic due to limited documentation during this time. In any case, by 1915, whaling became unprofitable in Icelandic waters as catch rates decreased and prices of whale oil inflated, majorly influenced by the increased availability and use of mineral oil. Iceland enforced a complete, two decades long ban on whaling at that time, before taking up new activities on a smaller scope. Owing to the uncertainty of fin whale catch numbers, the period without whaling, and because catch rates stayed on a relatively low and constant level since 1948 (Árnason 1981), it is possible that the bottleneck was not as severe compared to other areas of the world like in the waters of Finnmark, Norway, or compared with other whale species such as the blue whale (Tønnessen and Johnsen 1982).

By contrast, another whale species, the North Atlantic right whale, seems to show negative genetic effects from population depletion. Our genomic analyses revealed extensive ROH coverage and disproportionate, high levels of LoF mutations, notwithstanding high levels of heterozygosity. It is feared that there are <500 individuals remaining worldwide, which led to the classification as “critically endangered” on the IUCN red list (Cooke 2020). In addition, records of pre-industrial whaling (Tønnessen and Johnsen 1982), an increased anthropogenic mortality (Kraus et al. 2005) and slow reproduction rates (Browning et al. 2010) exist, indicating a long and persistent bottleneck for the species. High levels of genetic diversity contradicts previous findings based on microsatellites and mitochondrial marker but could potentially explained with what was previously reported by Frasier et al. (2013). The authors suggest that mating of genetically dissimilar individuals due to postcopulatory selection of gametes can lead to more heterozygous individuals compared to what would be expected by random mating. Although we cannot make definitive statements about this based on a single specimen, our finding of putatively substantial inbreeding opposes this assumption of non-random mating and furthermore contradicts the common assumption of the existence of a reciprocal relationship between population sizes, heterozygosity, inbreeding and mutational load which would lead to an “extinction vortex” as a consequence of a bottleneck that persisted for long periods of time (Fagan and Holmes 2006; Blomqvist et al. 2010; Teixeira and Huber 2021).

A correlation test of these parameters in all here analyzed baleen whales indicated no significant relationships between either of those factors. A nearly significant negative correlation was only identified between genome-wide heterozygosity and ROH, which is expected given their linked relationship. Nonetheless, the apparent absence of clear-cut relationships between effective population size, heterozygosity, ROH, and mutation load and IUCN status is consistent with previously reported findings. There is, for example, no significant correlation between the IUCN red list status of a population and their levels of inbreeding (measured by ROH) or genome-wide heterozygosity (Brüniche-Olsen et al. 2018) potentially induced by, for example, non-random mating of genetically dissimilar individuals as described in Frasier et al. (2013). There are also numerous examples of small populations with low genetic diversity and higher inbreeding that do not suffer from deleterious mutations due to purging, which further complicates the picture (Robinson et al. 2018; van der Valk et al. 2019b; Ochoa and Gibbs 2021). This suggests that if a population is heavily reduced, it might resist the reduction of genetic diversity or might resist the consequences of low genetic diversity. Therefore, we propose, similar to previous discussions (Teixeira and Huber 2021), that the reciprocal relationship between heterozygosity, inbreeding, and mutational load is not as direct as previously assumed and that measuring only one of those parameters could misjudge the actual level of endangerment of a population.

In the case of the analysis of the single genome of the North Atlantic right whale, this implies that their potentially high genetic diversity may not indicate a lowered risk of extinction. Instead and discussed earlier (van der Valk et al. 2019a), their increased levels of inbreeding and mutational load combined with a higher heterozygosity might even increase the risk of extinction disproportionally, because the potential higher number of deleterious mutations could become fixed more rapidly due to the emergence of ROH (van der Valk et al. 2019b; Teixeira and Huber 2021). To further evaluate the genetic risk in this species, population-genomic studies, like those presented here for the fin whale, are necessary to evaluate their population on a genomic level for targeted conservation efforts.

## Conclusion

Genome data of North Atlantic fin whales made it possible to assess the impact of whaling on the genetic diversity of a baleen whale population. Demographic analyses confirmed, consistent with historical records, that the population experienced a substantial reduction in its census size a century ago. Despite the decimation of their population and relatively low levels of heterozygosity compared to other whales, fin whales have a stable or even slightly increasing genome-wide diversity over time. In addition, there is no evidence for increased inbreeding or mutational load suggesting that the bottleneck, caused by whaling, had less impact on the genotype of the species as previously feared.

By contrast, analyses of other baleen whales revealed that the most threatened baleen whale species, the North Atlantic right whale, has relatively high levels of inbreeding and mutational load despite their potentially high genetic diversity. This calls for population-level genome-sequencing efforts for other baleen whales to enable a comprehensive conservation-genomic assessment and targeted conservation strategies.

## Materials and Methods

### Sampling, DNA Isolation, and Sequencing

A total of 51 tissue samples from individual fin whales were collected during fisheries operations in Icelandic waters in 1989, 2009, and 2018 (supplementary fig. S1, Supplementary Material online). The operation in 1989 was conducted under scientific research permit of the Icelandic Ministry of Food, Agriculture and Fisheries, operating in the years 1986–1989. The sampling in the years 2009–2018 was done during commercial fisheries operations licensed by the Icelandic Ministry of Food, Agriculture and Fisheries. Individual licenses are available on request.

Tissue samples were stored in 96% ethanol at −20°C and DNA was extracted from ∼20–50 mg tissue using a standard phenol-chloroform-isoamylalcohol protocol (Sambrook and Russell 2006). DNA libraries were prepared and sequenced by SciLifeLab, Stockholm, Sweden, or by Novogene, Cambridge, and United Kingdom. SciLifeLab and Novogene libraries were generated with the rubicon  thruPLEX dna-seq kit and the nebnext DNA library  prep kit, respectively, both according to manufacturer’s recommendations and using 350 bp insert size. Illumina short read sequencing was performed using the Illumina NovaSeq 6000 platform to produce 10-fold sequence coverage or ∼24 Gbp of 150 bp paired-end reads per individual. In addition, a single 10× Genomics Chromium library was compiled and sequenced by SciLifeLab, yielding 487,342,309 paired/linked 150 bp Illumina short reads (∼30-fold coverage).

### De novo assembly

A de novo genome of the fin whale was assembled using the linked short reads sequenced with the 10× chromium technology. We used supernova v2.1.1 (Weisenfeld et al. 2017) to construct pseudo-haplotype assemblies and evaluated their properties using quast v5.0.2 (Mikheenko et al. 2018). Additional scaffolding and correction steps were performed using arcs v1.1.1 (Yeo et al. 2018) and tigmint v1.1.2 (Jackman et al. 2018) which led to no substantial improvements, and we proceed using the best raw pseudo-haplotype assembly. The assembly was then assessed for coverage distribution (qualimap v2.2.2, Okonechnikov et al. 2016) and gene set completeness (busco v4.1.1, Seppey et al. 2019).

### Repeat and Genome Annotation

We screened the assembly for repetitive sequences using repeatmodeler v2 (www.repeatmasker.org) and merged found repeats with the *Cetartiodactyla* database from repbase (Jurka et al. 2005). The merged data set was used to mask repeats in our assembly using repeatmasker v4.1 (www.repeatmasker.org). Evidence and homology-based gene annotation was performed with the maker v2.31 pipeline (Holt and Yandell 2011) using data sets from the northern minke whale, *Balaenoptera acutorostrata* (Yim et al. 2014). Furthermore, genes were predicted using augustus v3.2.2 (Stanke et al. 2006) and genemark-ES v4 (Lomsadze et al. 2005) as implemented in maker. Finally, we annotated gene functions to the predicted protein sequences using interproscan v5 (Jones et al. 2014) with default parameters.

### Read Mapping and SNP Calling/Filtering

Short read sequences were examined using fastQC v0.11.8 (https://www.bioinformatics.babraham.ac.uk/projects/fastqc/) and trimmed for read quality with fastp (Chen et al. 2018) and for adapter sequences with adapterremoval v2 (Schubert et al. 2016). Trimmed reads were mapped to the de novo assembled fin whale genome using bwa-mem v0.7.17-r1188 (http://bio-bwa.sourceforge.net). Potential duplicates were removed, and read-groups were added using the picard  v2.21.2-0 toolkit (https://broadinstitute.github.io/picard/). Genotypes including multi-variant and monomorphic sites were called in a combined approach including all mapping files as well as individually per single mapping file to account for different needs of downstream analyses. This was done with bcftools  v1.12 mpileup and bcftools  v1.12 call (Danecek et al. 2021) using the “-m” or “-c” flags respectively applying a minimal mapping- and base-quality cutoffs of 30 using the flags “-q” and “-Q”. bcftools  v1.12 filter (Danecek et al. 2021) was used to exclude indels, sites with divergent read coverage (>3-fold and <0.3-fold of the expected mean coverage) and sites with more than 5% missing data. In the case of the combined data set, vcftools  v0.1.16 (Danecek et al. 2011) was used to remove multi-variants sites as well to retrieve SNPs. In addition, for the combined data set, plink v1.90p (Chang et al. 2015) was used with “–indep-pairwise 1,000 kb 1 0.9” parameters to remove sites in potential linkage disequilibrium. Furthermore, putatively related individuals were removed after an identity-by-descent test using the “–genome” function of plink v1.90p (Chang et al. 2015) applying an pi_hat cutoff of 0.2. The final combined dataset consists of 966,242,959 genotypes and 7,022,898 SNPs whilst the individually called data sets contain between 976 Mio and 1 Bio genotypes. To compare our findings against other whale species, these steps were repeated by mapping raw reads of five different baleen whale species (Árnason et al. 2018) sequenced with the same sequencing platform like used in this study. All five genome data sets and the here presented fin whale genomes were mapped against the bowhead whale, *Balaena mysticetus,* reference genome (Keane et al. 2015). To ensure comparability, we repeated each step, starting from quality filtering of raw reads up to the filtering of single nucleotide variances (SNVs), all with equal filter parameters. This second combined data set consists of 56 individuals, six baleen whale species, 469,467,070 genotypes, and 14,857,736 SNVs. Individually called data sets contain between 115 Mio and 1 Bio genotypes.

### Genetic Structure and Population Differentiation Analysis

We divided the fin whale samples into multiple cohorts based on their capture years: 1989, 2009, and 2018. Population structure analyses were then performed on the combined fin whale SNP data set that was further thinned randomly (one SNP per 1 kbp) with vcftools  v0.1.16 thin (Danecek et al. 2011) to reduce the computational load of the following steps. The R-package sambaR (de Jong et al. 2021) was used to filter out individuals with more than 5% missing data as well as SNPs with more than 10% missing data, heterozygosity excess, and a minor allele count of 1. Population-genetic analyses were performed by using sambaR’s main functions “findstructure()” and “calcdistance()”. Among the analyses invoked by these wrapper functions are PCoA performed with the ape-5.3 package (Paradis and Schliep 2019) and admixture analysis performed with the LEA-2.4.0 package (Frichot et al. 2015).

### Genetic Diversity

Nucleotide diversity, Watterson’s *θ* and Tajima’s *D* estimates were generated using sambaR’s main function “calcdiversity()”. Genome-wide heterozygosity was inferred by counting heterozygous sites in the individual VCF files that still included multi-variant and monomorphic sites. To test for potential significant differences in genome-wide heterozygosity between the three cohorts, an ANOVA test was conducted. A fSFS was generated using the “vcf_to_sfs” tool distributed within the popgen  pipeline  platform (Webb et al. 2021).

### Demographic Analysis

Demographic history of the fin whale population was inferred based on the fSFS using the Java package stairway plot v2 (Liu and Fu 2020) which estimates series of mutations rates over time. For all demographic estimations, we defined a mutation rate of 1.54 × 10^−9^ per site per generation following Tollis et al. (2019) and a generation time of 25.9 years following Taylor et al. (2007). We determined the modeled time window to the last 800 years (∼30 generations) and conducted analyses based on the fSFS of the total fin whale sampling as well as on the fSFS of the individual cohorts (fig 1).

To further elucidate the role of different demographic events on the fSFS, we implemented forward-in-time Wright-Fischer simulations using sliM v3.7 (Haller and Messer 2019) to generate expected fSFS given a certain scenario (supplementary table S4, Supplementary Material online). For each simulation, we started with a population of 50,000 individuals and three 10 Mbp long genomic elements. The mutation rate was set to 1.54 × 10^−9^ per site per generation and the recombination rate was defined as 1 × 10^−8^ per generation. Each simulation ran without any events for 100,000 generations to obtain neutral fSFS. Afterwards, demographic changes were applied as depicted in supplementary figure S3*A*–*H*, Supplementary Material online and a number of individuals were sampled equal to the number of fin whale individuals. fSFS were extracted from the resulting vcf files using the “vcf_to_sfs” tool as described above (Webb et al. 2021). Eventually, we compared different demographic simulations by calculating log-likelihoods of observing the empirical fSFS given one of the simulated fSFS using the R base function “dmultinom” (supplementary table S4, Supplementary Material online).

Additionally, we used the PSMC framework (Li and Durbin 2011) to model historical *N*_e_ further back in time (10 kya to 1 Mya) using the individual vcf files constructed with the fin whale reference genome as described before. These files were filtered as described above before inferring consensus sequences with bcftool’s vcfutils.pl. Consensus sequences were then used for the PSMC modeling using the same mutation rate and generation time as for the stairway plot analysis (supplementary fig. S5, Supplementary Material online).

### Inbreeding Estimation and ROH

Inbreeding factors based on the excess of homozygous sites were estimated for the complete fin whale dataset only as it requires assumptions about the expected number of heterozygous sites per population (supplementary fig. S6, Supplementary Material online), whereas ROHs were collected for all individual vcf files including the data of other baleen whale species. By comparing proportions of observed and expected homozygous sites using sambaR’s “clackinship” function, inbreeding coefficients for all three cohorts were gathered (*F*_H_, supplementary fig. S6, Supplementary Material online, Kardos et al. 2015). ROHs were identified with darwindow (https://github.com/mennodejong1986/Darwindow), which finds ROH per individual with a sliding window approach. ROHs were detected using a sliding window size of 10 kbp, a heterozygosity threshold of 0.2%, and a minimal window number of 10. Excluded from the analysis were scaffolds with a size below 3 Mbp. Found ROH were subsequently sorted into different length bins ranging from 100 kbp to over 1 Mbp with a step size of 100 kbp. Detailed graphs with bins from 100 kbp to over 4 Mbp are depicted in supplementary figure S7, Supplementary Material online. Individual inbreeding coefficients per bin were calculated from the extent of ROH spanning the respective reference genome (*F*_ROH_, after McQuillan et al. 2012) and an ANOVA test was conducted to find significant differences between *F*_ROH_ of the different fin whale cohorts (supplementary fig. S8, Supplementary Material online). Potential correlations were estimated between genome-wide HE and either *F*_ROH_ or *F*_H_ in R using a Pearson correlation test.

### Mutational Load

Mutational load was inferred based on the functional annotation of variants. SNPs and SNVs were assigned with potential functional categories with snpeff v4.3 (Cingolani et al. 2012) using the annotation generated in this study and the annotation generated by Keane et al. (2015). To get the total number of variants, individual vcf files were filtered as described above before annotating them with snpeff using default parameters. Resulting functionally assigned variants were sorted into the categories *synonymous*, *missense,* and *loss of function* (LoF), and normalized using the total number of variants per individual. Mutational load was defined as the proportion of LoF mutation compared with the respective total counts of variances. The numbers of LoF mutations were furthermore differentiated between heterozygous and homozygous variants to estimate which proportion might actually affect the fitness of the individual. Finally, potential correlations between either the total number of LoF mutations or the relative abundance (mutational load) were inferred from genome-wide HE and *F*_ROH_ applying a standard Pearson correlation test in R.

## Supplementary Material

Supplementary data are available at *Molecular Biology and Evolution* online.

## Supplementary Material

msac094_Supplementary_Data

## Data Availability

Raw sequencing reads have been deposited at the National Center for Biotechnology Information under the BioProject PRJNA740292. The assembled genome sequence of the fin whale is deposited as Genome: JAHXJN000000000, BioSample: SAMN19897699. All other data needed to evaluate the conclusions of the article are present in the paper and/or the Supplementary Materials. Additional data related to this paper may be requested from the authors.
