# Supplementary material for: Genomic Impact of Whaling in North Atlantic Fin Whales
Source: Mol Biol Evol. 2022 May 5;39(5):msac094. doi: 10.1093/molbev/msac094 (PMC9113106; doi:10.1093/molbev/msac094)
Supplement: msac094_Supplementary_Data [file msac094_supplementary_data.docx]

Supplementary Materials for

**Genomic impact of whaling in North Atlantic fin whales.**

Magnus Wolf, Menno de Jong, Sverrir Daníel Halldórsson, Úlfur Árnason, Axel Janke

*Corresponding author: Magnus Wolf; Email: [Magnus.Wolf@senckenberg.de](mailto:Magnus.Wolf@senckenberg.de)

**This PDF file includes:**

Figs. S1 to S8

- **Fig. S1** Geographical distribution of sampled whales presented in this study.
- **Fig. S2** Folded site frequency spectrum for the three fin whale cohorts sampled in total,1989, 2009, and 2018, respectively.
- **Fig. S3** Demographic scenarios simulated with forward-in-time Wright-Fischer model simulations using SLiM, arranged in order of model performance.
- **Fig. S4** Comparison between the empirical fSFS and the fSFS predicted by forward-in-time Wright-Fischer model simulations using SLiM, arranged in order of model performance (i.e. log-likelihood score).
- **Fig. S5** Changes in N_e_ over the last 1 Mya to 10 kya compared among the fin whale cohorts using a PSMC analysis.
- **Fig. S6** Distribution of inbreeding coefficients F_H_ (Kardos et al. 2015) for the three fin whale cohorts sampled in the years 1989 (red), 2009 (blue) and 2018 (green).
- **Fig. S7** Inbreeding factors (F_ROH_) based on the genome coverage of run of homozygosity (ROH) between different minimal lengths cutoffs of ROH: 100 kbp to over 4 Mbp (x-axis in 100 kbp steps).
- **Fig. S8** ANOVA significance test comparing inbreeding coefficient of three fin whale cohorts based on runs of homozygosity longer than 1 Mbp.

Tables S1 to S4

- **Table S1** General information of sampled fin whales that were sequenced and analyzed in this study.
- **Table S2** Summary statistics, BUSCO completeness analyses and annotation statistics for the fin whale reference genome.
- **Table** **S3** Repeat content of the fin whale assembly compiled for this study.
- **Table** **S4** Log-likelihood scores showing the probability of observing the empirical fSFS given the predicted fSFS for each forward-in-time Wright-Fischer model simulation using SLiM.

References used in this document are all included in the main manuscript.

**Supplementary Figures**


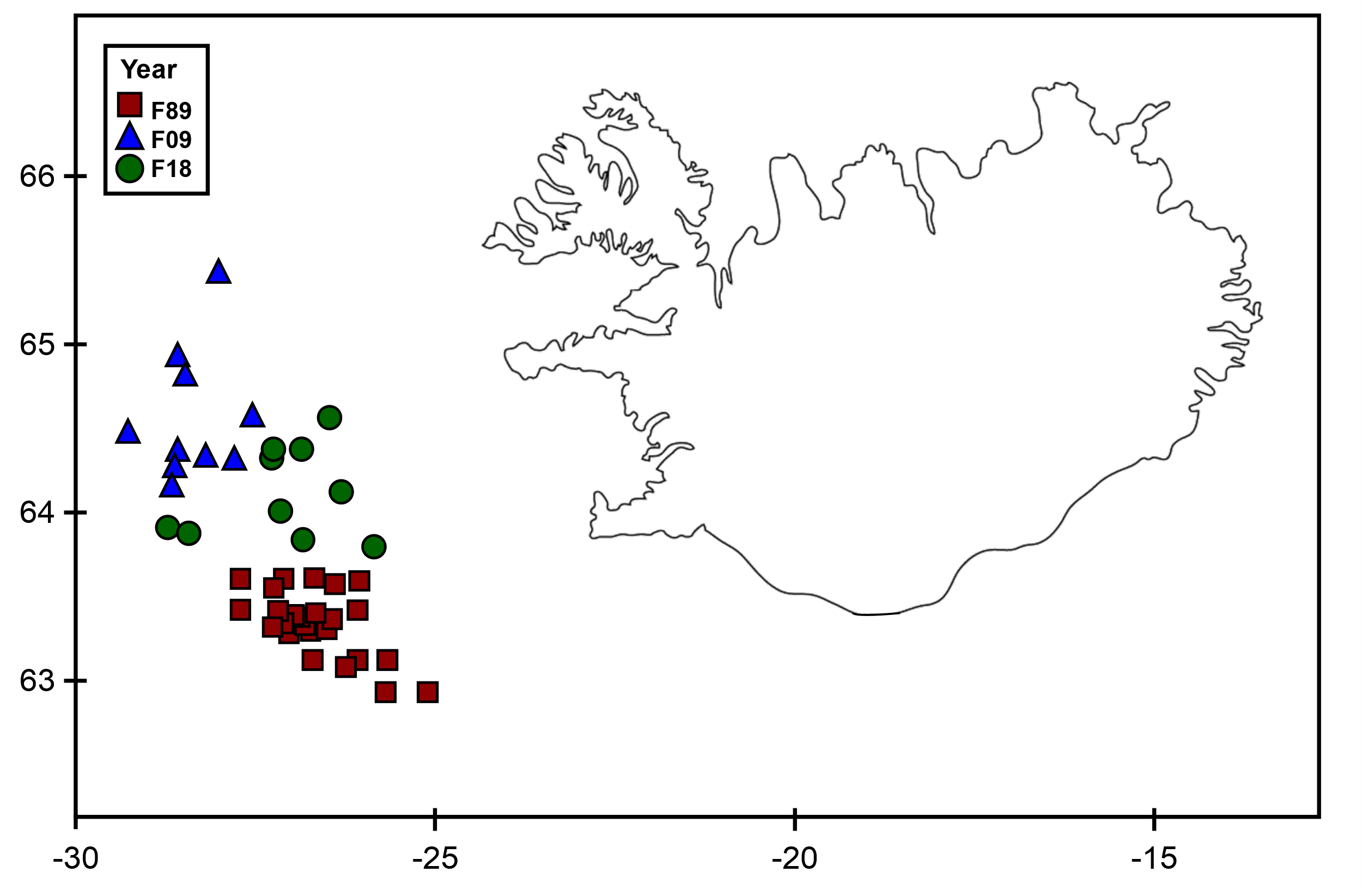


**Fig. S1** Geographical distribution of sampled whales presented in this study. F and the following number in the upper left box denotes the sampling year (1989: red, 2009: blue, 2018: green) of a fin whale (F). Numbers on the coordinates denote the latitude and longitude (WGS84), shown in Table S1.


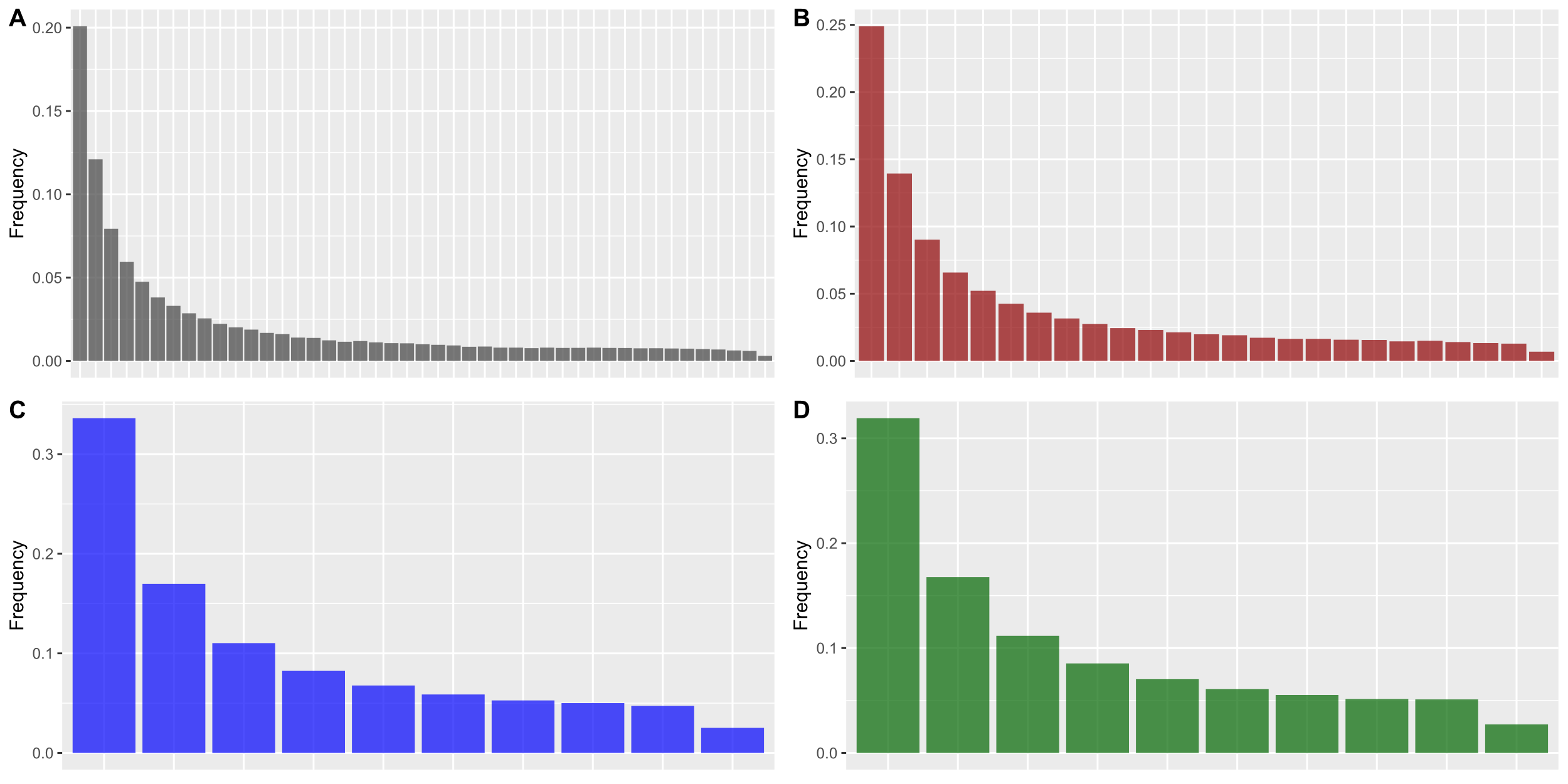


**Fig. S2** Folded site frequency spectrum (fSFS) for all fin whale samples combined (A, grey) and the three fin whale cohorts 1989 (B, red), 2009 (C, blue) and 2018 (D, green) separately. Bars indicate the proportion of polymorphic sites for a given number of minor allele copies. fSFS were used for downstream analyses like the demographic models estimated by Stairway Plot v2 (Liu and Fu 2020).


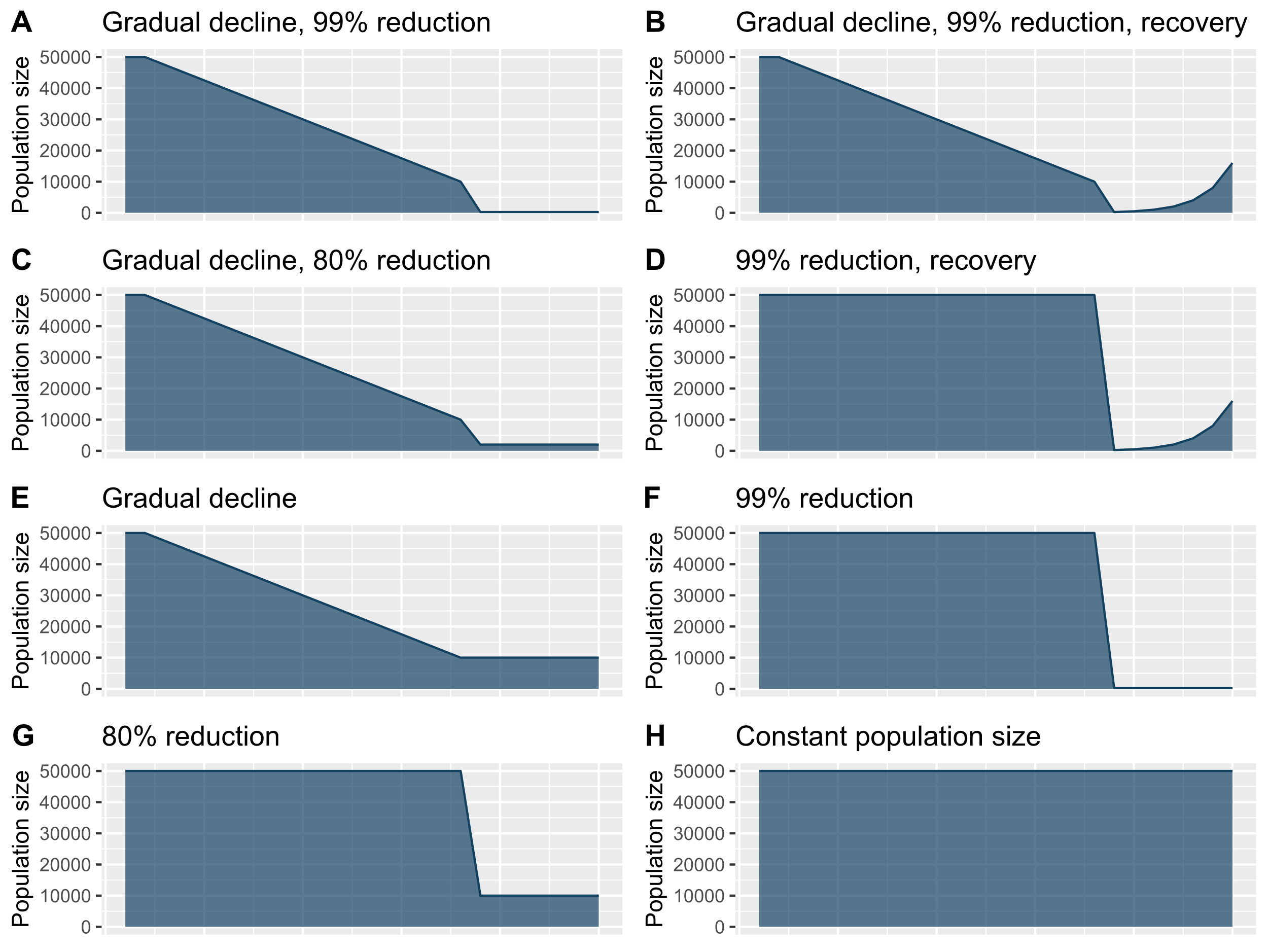


**Fig. S3** Demographic scenarios simulated with forward-in-time Wright-Fischer model simulations using SLiM (Haller et al. 2019), arranged in order of model performance (Table S4). Model performance was estimated as the log-likelihood of observing the empirical fSFS given the simulated fSFS. Demographic scenarios involving migration between a bottlenecked and a non-bottlenecked population performed poorly and have been omitted here.


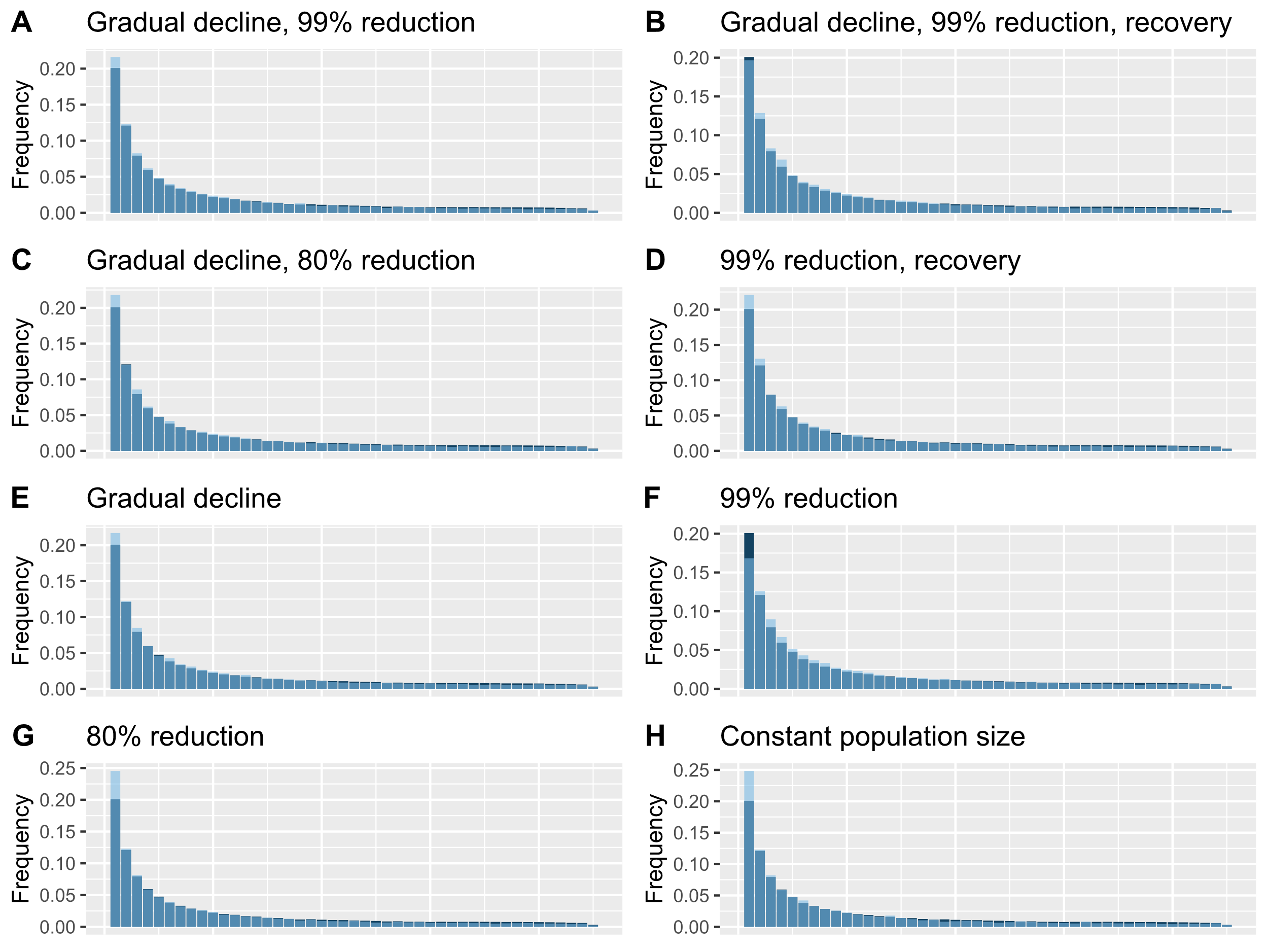


**Fig. S4** Comparison between the empirical fSFS (Fig. S2) and the fSFS predicted by forward-in-time Wright-Fischer model simulations using SLiM (Haller et al. 2019), arranged in order of model performance (i.e., log-likelihood score, Table S4). Darkblue: empirical fSFS; lightblue predicted fSFS.


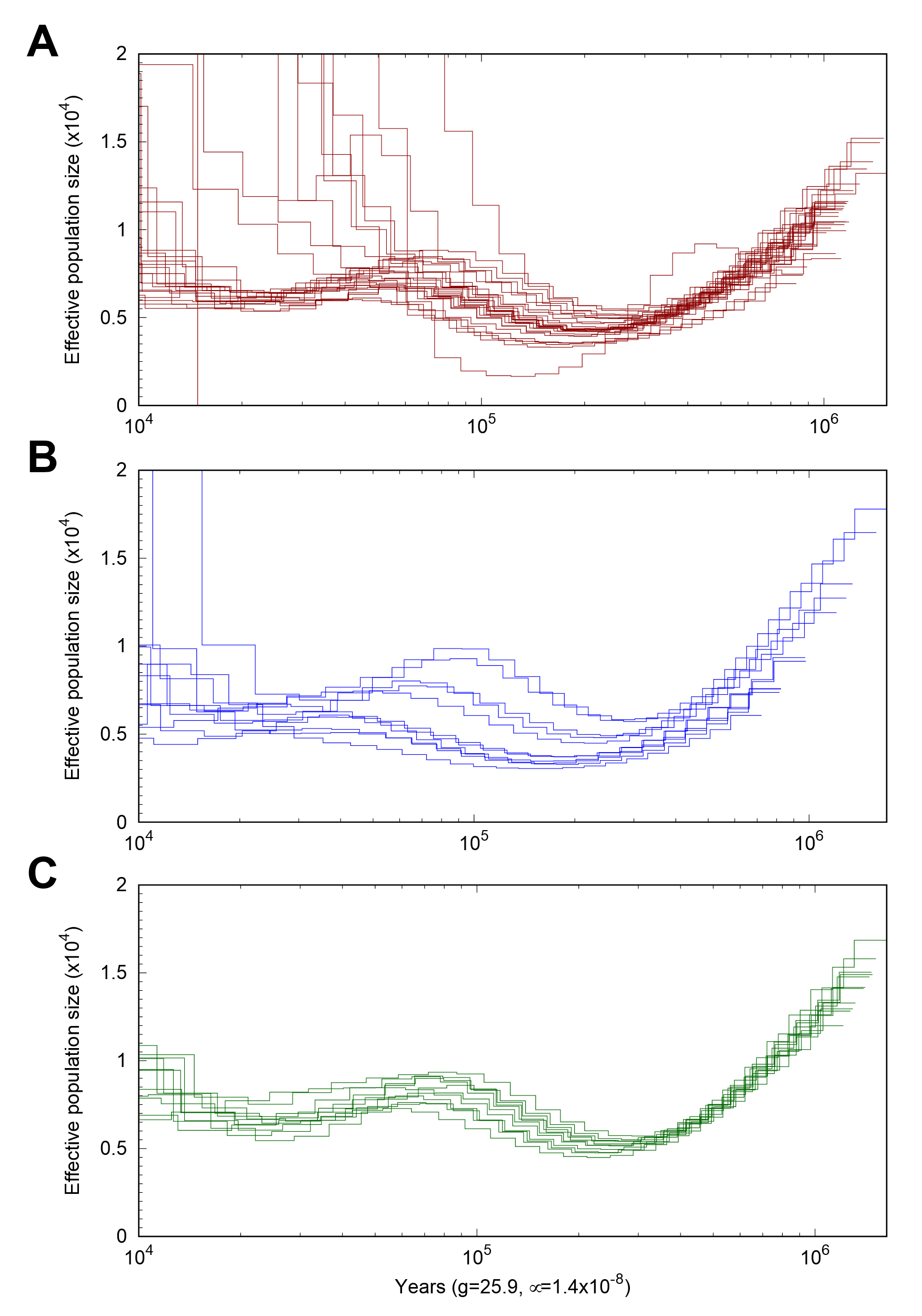


**Fig. S5** Changes in N_e_ over the last 1 Mya to 10 kya compared among the fin whale cohorts (color code matches main manuscript) using a PSMC analysis (Li and Durbin 2011). For the three cohorts, the trajectory follows the results shown in Árnason et al. (2018), showing a rather stable, slightly fluctuating N_e_ over time. Plots were scaled using a mutation rate of 1.54 x 10^-9^ per site per generation and a generation time of 25.9 years.


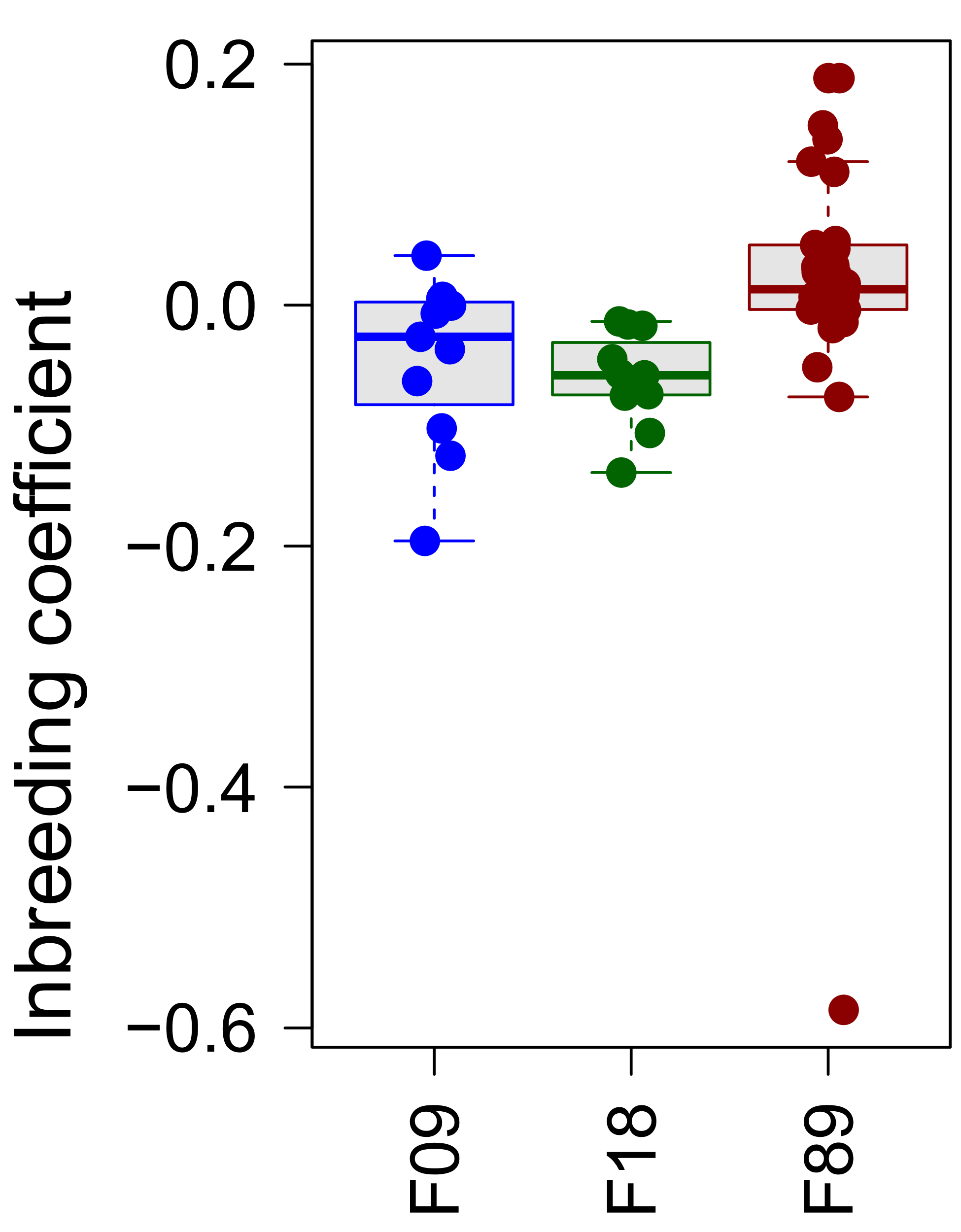


**Fig. S6** Distribution of inbreeding coefficients F_H_ (Kardos et al. 2015) for the three fin whale cohorts sampled in the years 1989 (red), 2009 (blue) and 2018 (green). The mean value was slightly negative for all cohorts. However, in the 2009 and 2018 cohort, there were more negative inbreeding factors suggesting slight excess in heterozygous genotypes compared to the expected value zero.


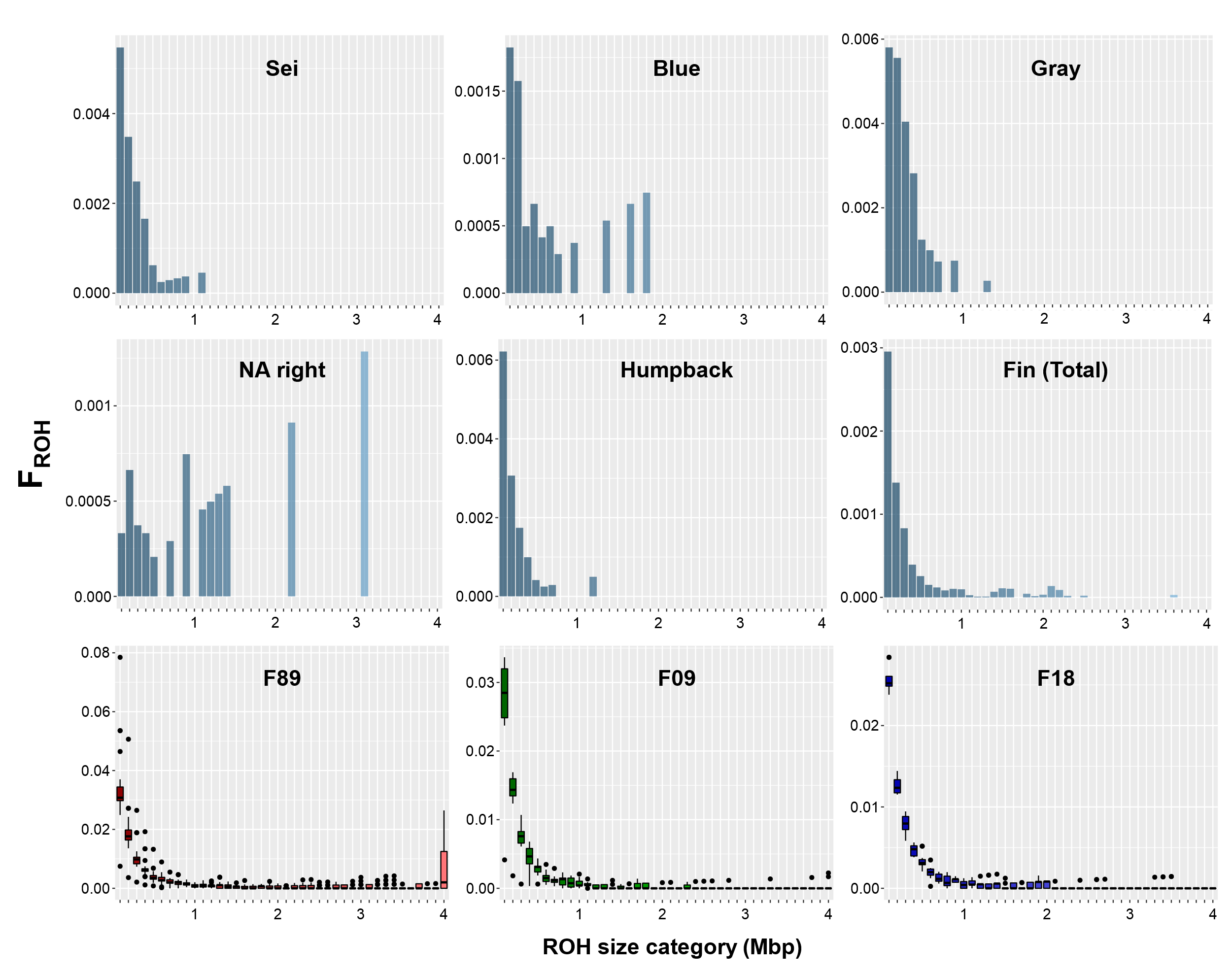


**Fig. S7** Inbreeding factors (F_ROH_) based on the genome coverage of run of homozygosity (ROH) between different minimal lengths cutoffs of ROH: 100 kbp to over 4 Mbp (x-axis in 100 kbp steps).


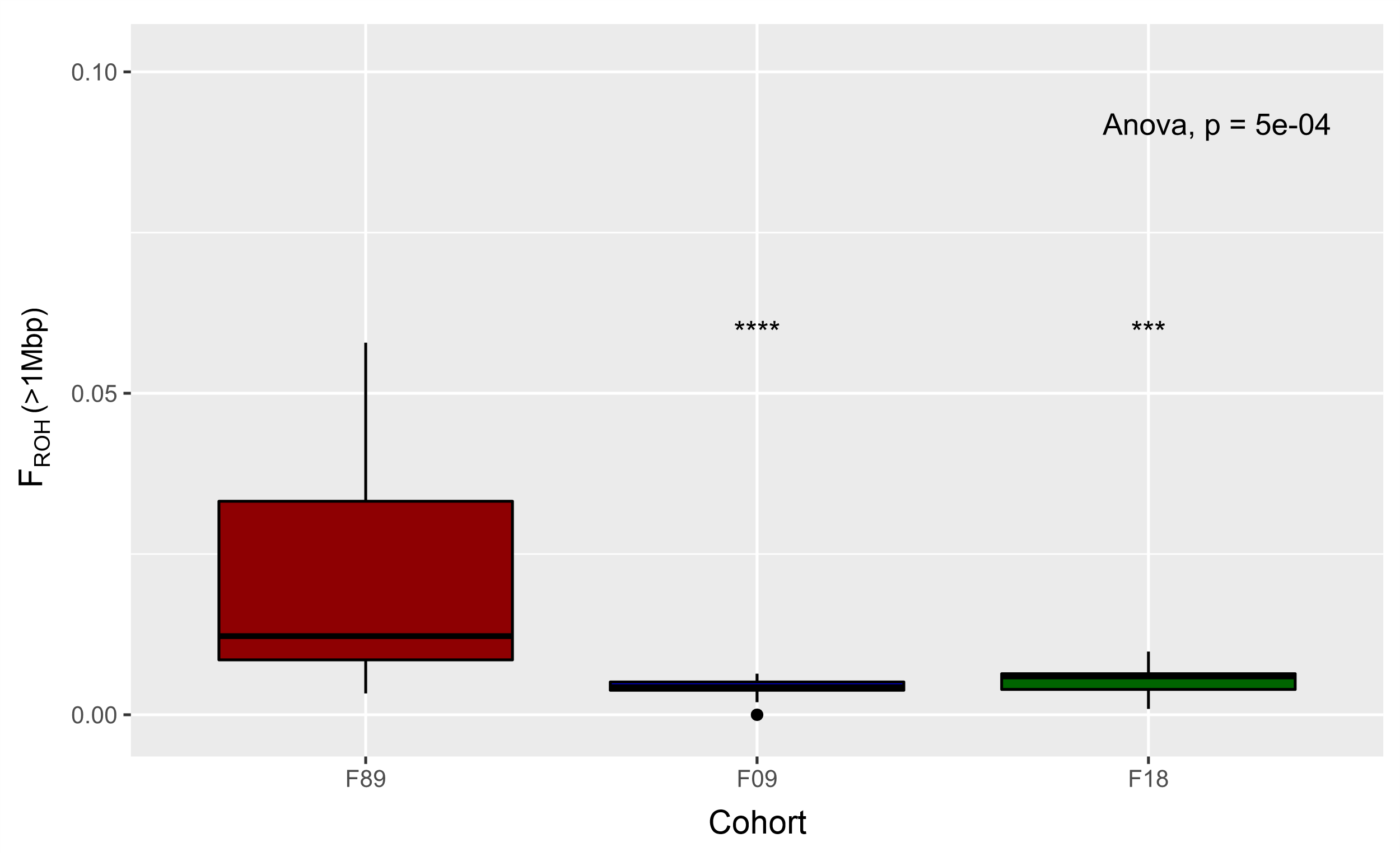


**Fig. S8** ANOVA significance test comparing inbreeding coefficients (F_ROH_) of three fin whale cohorts (1989: red, 2009: blue, 2018: green) based on runs of homozygosity longer than 1 Mbp. The total proportion of the reference genome covered by those ROH varied significantly between 1989 and both other cohorts (2009: *p=0.003*; 2018: *p=0.006*), respectively. Between the 2009 and 2018 cohort, no significant differences were found. Following these results, it can be assumed that some individuals of the 1989 cohort experienced inbreeding in the more recent past.

**Supplementary Tables**

**Table S1** General information of sampled fin whales that were sequenced and analyzed in this study. Included are the used sample-ID, sex as well as the specific capture location and date**.**

| **Sample-ID** | **Sex** | **Latitude** | **Longitude** | **Date** |  | **Sample-ID** | **Sex** | **Latitude** | **Longitude** | **Date** |
| --- | --- | --- | --- | --- | --- | --- | --- | --- | --- | --- |
| **F89001** | **f** | **63.6** | **26.2** | **19. Jun. 89** |  | **F09019** | **f** | **65.4** | **28.1** | **03. Jul 09** |
| **F89002** | **f** | **63.6** | **27.8** | **21. Jun. 89** |  | **F09020** | **m** | **64.6** | **27.6** | **04. Jul 09** |
| **F89003** | **m** | **63.4** | **27.8** | **21. Jun. 89** |  | **F09021** | **f** | **64.3** | **28.3** | **05. Jul 09** |
| **F89004** | **f** | **63.1** | **26.8** | **21. Jun. 89** |  | **F09022** | **m** | **64.2** | **28.7** | **05. Jul 09** |
| **F89005** | **f** | **63.4** | **27.2** | **21. Jun. 89** |  | **F09023** | **m** | **64.3** | **28.7** | **06. Jul 09** |
| **F89006** | **f** | **63.6** | **26.8** | **22. Jun. 89** |  | **F09024** | **f** | **64.2** | **28.7** | **06. Jul 09** |
| **F89007** | **f** | **63.6** | **26.8** | **23. Jun. 89** |  | **F09025** | **m** | **64.8** | **28.6** | **08. Jul 09** |
| **F89008** | **f** | **63.6** | **26.5** | **23. Jun. 89** |  | **F09026** | **f** | **64.9** | **28.6** | **08. Jul 09** |
| **F89009** | **f** | **63.4** | **26.8** | **23. Jun. 89** |  | **F09027** | **m** | **64.3** | **27.9** | **09. Jul 09** |
| **F89010** | **f** | **63.4** | **26.8** | **24. Jun. 89** |  | **F09028** | **m** | **64.5** | **29.3** | **10. Jul 09** |
| **F89011** | **f** | **63.4** | **26.8** | **24. Jun. 89** |  | **F09029** | **f** | **64.5** | **29.4** | **10. Jul 09** |
| **F89012** | **f** | **63.4** | **27.2** | **24. Jun. 89** |  | **F18003** | **m** | **64.4** | **26.9** | **24.Jun 18** |
| **F89013** | **m** | **63.4** | **26.8** | **24. Jun. 89** |  | **F18004** | **f** | **64.1** | **26.4** | **24.Jun 18** |
| **F89014** | **f** | **63.4** | **26.8** | **25. Jun. 89** |  | **F18006** | **f** | **64.4** | **27.4** | **28. Jun 18** |
| **F89015** | **m** | **63.4** | **26.8** | **25. Jun. 89** |  | **F18007** | **m** | **64.3** | **27.4** | **28. Jun 18** |
| **F89016** | **m** | **63.4** | **26.8** | **25. Jun. 89** |  | **F18008** | **f** | **64.6** | **26.6** | **29. Jun 18** |
| **F89017** | **f** | **63.1** | **26.8** | **26. Jun. 89** |  | **F18009** | **m** | **63.9** | **28.5** | **30. Jun 18** |
| **F89018** | **f** | **63.4** | **26.2** | **27. Jun. 89** |  | **F18010** | **f** | **63.9** | **28.8** | **30. Jun 18** |
| **F89019** | **m** | **63.4** | **26.2** | **27. Jun. 89** |  | **F18011** | **m** | **64.0** | **27.2** | **03. Jul 18** |
| **F89020** | **f** | **63.6** | **27.2** | **27. Jun. 89** |  | **F18013** | **f** | **63.8** | **26.0** | **03. Jul 18** |
| **F89030** | **m** | **62.9** | **25.2** | **1. Jul. 89** |  | **F18014** | **m** | **63.8** | **26.0** | **03. Jul 18** |
| **F89031** | **m** | **62.9** | **25.8** | **1. Jul. 89** |  | **F18015** | **m** | **63.8** | **26.9** | **05. Jul 18** |
| **F89032** | **m** | **62.9** | **25.8** | **1. Jul. 89** |  |  |  |  |  |  |
| **F89033** | **f** | **63.1** | **25.8** | **2. Jul. 89** |  |  |  |  |  |  |
| **F89035** | **f** | **63.1** | **26.8** | **3. Jul. 89** |  |  |  |  |  |  |
| **F89036** | **f** | **63.4** | **26.8** | **3. Jul. 89** |  |  |  |  |  |  |
| **F89037** | **f** | **63.1** | **26.2** | **3. Jul. 89** |  |  |  |  |  |  |
| **F89038** | **m** | **63.1** | **26.2** | **3. Jul. 89** |  |  |  |  |  |  |

f, female; m, male.

**Table S2** Summary statistics, BUSCO completeness analyses and annotation statistics for the fin whale reference genome.

| **Assembly statistics** | | |
| --- | --- | --- |
| **No. contigs** |  | **13,639** |
| **No. contigs (>50 kbp)** |  | **305** |
| **L50** |  | **27** |
| **L75** |  | **58** |
| **N50 (bp)** |  | **24,939,677** |
| **N75 (bp)** |  | **14,958,115** |
| **Max.contig length (bp)** |  | **91,471,248** |
| **Total length (bp)** |  | **2,412,335,827** |
| **GC (%)** |  | **40.81** |
| **No. of N's per 100 kbp** |  | **1,702.22** |
| **BUSCO completeness** | | |
| **BUSCO (*Cetartiodactyla*)** |  | **C:83.4%[S:82.1%,D:1.3%],** |
|  |  | **F:4.1%,M:12.5%,** |
|  |  | **n:13335** |
| **BUSCO (*Laurasiatheria*)** |  | **C:90.5%[S:89.0%,D:1.5%]** |
|  |  | **F:3.0%,M:6.5%** |
|  |  | **n:12234** |
| **BUSCO (*Mammalia*)** |  | **C:91.2%[S:89.9%,D:1.3%]** |
|  |  | **F:2.5%,M:6.3%** |
|  |  | **n:9226** |
| **Annotation statistics** | | |
| **Total interspersed repeats (bp)** |  | **1,008,206,033 (41.79 %)** |
| **Number of transcripts** |  | **17,307** |
| **Functional annotated genes** |  | **17,152 (99.1%)** |

BUSCO: Benchmarking Universal Single Copy Orthologs (Seppey et al. 2019);

C, complete; S, single copy; D, duplicated; F, fragmented; M, missing.

**Table** **S3** Repeat content of the fin whale assembly compiled for this study. Repeats were collected by Repeatmasker v4.1 ([www.repeatmasker.org](http://www.repeatmasker.org)) after modelling and identifying them with Repeatmodeler v2 ([www.repeatmasker.org](http://www.repeatmasker.org)) using the Cetartiodactyla database from RepBase (Jurka et al. 2005).

|  | Number of elements | Length occupied (bp) | Percentage of sequence |
| --- | --- | --- | --- |
| Retroelements | 2286384 | 918589536 | 38.08 |
| SINEs: | 666521 | 121878092 | 5.05 |
| Penelope | 65 | 13180 | 0.00 |
| LINEs: | 1235730 | 649137675 | 26.91 |
| CRE/SLACS | 0 | 0 | 0.00 |
| L2/CR1/Rex | 254400 | 65703824 | 2.72 |
| R1/LOA/Jockey | 0 | 0 | 0.00 |
| R2/R4/NeSL | 402 | 105485 | 0.00 |
| RTE/Bov-B | 10799 | 3251621 | 0.13 |
| L1/CIN4 | 969766 | 579940256 | 24.04 |
| LTR elements: | 384133 | 147573769 | 6.12 |
| BEL/Pao | 0 | 0 | 0.00 |
| Ty1/Copia | 1086 | 2740340 | 0.11 |
| Gypsy/DIRS1 | 14835 | 4166645 | 0.17 |
| Retroviral | 357392 | 137801181 | 5.71 |
| DNA transposons: | 384998 | 84056461 | 3.48 |
| hobo-Activator | 274224 | 55671684 | 2.31 |
| Tc1-IS630-Pogo | 97076 | 26618088 | 1.10 |
| En-Spm | 0 | 0 | 0.00 |
| MuDR-IS905 | 0 | 0 | 0.00 |
| PiggyBac | 631 | 216795 | 0.01 |
| Tourist/Harbinger | 334 | 60928 | 0.00 |
| Other  (Mirage,Pelement,Transib) | 0 | 0 | 0.00 |
|  |  |  |  |
| Rolling-circles | 1597 | 407845 | 0.02 |
| Unclassified: | 29118 | 5560036 | 0.23 |
| Total interspersed repeats: |  | 1008206033 | 41.79 |
| Small RNA: | 292610 | 66915824 | 2.77 |
| Satellites: | 3649 | 1767591 | 0.07 |
| Simple repeats: | 12507 | 2299460 | 0.10 |
| Low complexity: | 0 | 0 | 0.00 |

**Table** **S4** Log-likelihood scores showing the probability of observing the empirical fSFS given the predicted fSFS for each forward-in-time Wright-Fischer model simulation using SLiM (Haller et al. 2019). Log-likelihood scores have been calculated using the R base function ‘dmultinom’.

| Scenario | Previous Demography | Whaling Decimation | Following Demography | Migration | Log-Likelihood |
| --- | --- | --- | --- | --- | --- |
| 1 | Gradual decline | 99% | - | - | -1.207,963 |
| 2 | Gradual decline | 99% | Recovery | - | -1.327,108 |
| 3 | Gradual decline | 80% | - | - | -1.327,108 |
| 4 | - | 99% | Recovery | - | -1.350,702 |
| 5 | Gradual decline | - | - | - | -1.474,814 |
| 6 | - | 99% | - | - | -1.773,815 |
| 7 | - | 80% | - | - | -2.353,175 |
| 8 | - | - | - | - | -3.039,545 |
| 9 | Gradual decline | 99% | - | slow Migration | -10.530,82 |
| 10 | - | 99% | - | fast Migration | -10.717,62 |
| 11 | Gradual decline | 99% | - | fast Migration | -11.776,07 |
| 12 | - | 80% | - | slow Migration | -11.969,25 |
| 13 | Gradual decline | 99% | Recovery | fast Migration | -13.797,7 |
| 14 | Gradual decline | 80% | - | slow Migration | -14.187,21 |
| 15 | Gradual decline | - | - | slow Migration | -14.914,1 |
| 16 | Gradual decline | 80% | - | fast Migration | -15.270,46 |
| 17 | Gradual decline | - | - | fast Migration | -15.565,79 |
| 18 | - | 80% | - | fast Migration | -17.072,05 |
| 19 | - | - | - | slow Migration | -17.599,82 |
| 20 | - | 80% | - | slow Migration | -18.100,24 |
| 21 | - | - | - | fast Migration | -18.368,42 |
